# Isoliensinine suppressed gastric cancer cell proliferation and migration by targeting TGFBR1 to regulate TGF-β-smad signaling pathways

**DOI:** 10.3389/fphar.2024.1438161

**Published:** 2024-09-19

**Authors:** Jinda Hu, Shangming Dai, Mengqin Yuan, Fengjiao Li, Shuoguo Xu, Lichen Gao

**Affiliations:** ^1^ Department of Pharmacy, School of Pharmacy, Phase I Clinical Trial Centre, The Affiliated Changsha Central Hospital, Hengyang Medical School, University of South China, Changsha, China; ^2^ Hunan Provincial Key Laboratory of Tumor Microenvironment Responsive Drug Research, Hengyang, China

**Keywords:** gastric cancer, isoliensinine, epithelial-mesenchymal transition (EMT), metastasis, proliferation, transforming growth factor-β (TGF-β)

## Abstract

**Background:**

Gastric cancer (GC) ranks as the fifth most prevalent cancer globally, and its pronounced invasiveness and propensity to spread provide significant challenges for therapy. At present, there are no efficacious medications available for the treatment of patients with GC. Isoliensinine (ISO), a bisbenzylisoquinoline alkaloid, was isolated from *Nelumbo nucifera* Gaertn. It possesses anti-tumor, antioxidant, and other physiological effects. Nevertheless, there is currently no available study on the impact of ISO on GC, and further investigation is needed to understand its molecular mechanism.

**Methods:**

ISO target points and GC-related genes were identified, and the cross-target points of ISO and GC were obtained. We then examined cross-targeting and found genes that were differentially expressed in GCs. Kaplan-Meier survival curves were used to screen target genes, and the STRING database and Cytoscape 3.9.1 were used to construct protein-protein interactions and drug-target networks. In addition, molecular docking studies confirmed the interactions between ISO screen targets. Finally, *in vitro* tests were used to establish the impact of ISO on GC cells.

**Results:**

Through bioinformatics research, we have identified TGFBR1 as the target of ISO in GC. In addition, we noticed a substantial inhibition in GC cell proliferation, migration, and invasion activities following ISO treatment. Moreover, we noticed that ISO treatment effectively suppressed TGF-β-induced epithelial-mesenchymal transition (EMT) and activation of the TGF-β-Smad pathway. Furthermore, we discovered that siTGFBR1 nullified the impact of ISO on TGF-β-triggered migration, invasion, and activation of the TGF-β-Smad pathway.

**Conclusion:**

Our research suggests that ISO specifically targets TGFBR1 and regulates the TGF-β-Smad signaling pathway to suppress the proliferation and migration of GC cells.

## Graphical Abstract

**Figure:**
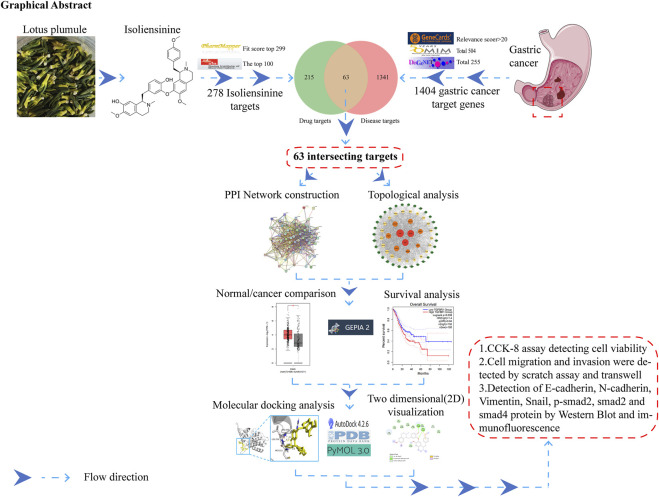
The study’s flow chart utilizes network pharmacology, molecular docking, and experimental verification approaches to predict the anti-GC activity of ISO.

## 1 Introduction

Gastric cancer (GC) is a prevalent and severe malignancy that has consistently been a prominent issue in global public health (Ajani et al., 2022). Despite considerable advancements in recent decades, treating GC remains challenging due to its highly aggressive character and potential to metastasize (Li et al., 2022a). The treatment of advanced stages of GC, in particular, poses significant difficulties (Tan, 2019). Hence, the identification of novel therapeutic approaches and targets is a crucial focus in contemporary research on GC (Guan et al., 2023).

ISO is a kind of bisbenzylisoquinoline alkaloid obtained from the lotus seeds’ core. It comprises two benzylisoquinolines linked by an ether bond (Zhang et al., 2015). The lotus seed heart’s primary purpose, as stated in the 2020 edition of the “Chinese Pharmacopoeia,” the lotus seed heart’s primary purpose is to alleviate anxiety and promote tranquility of the mind (Pei et al., 2021). These findings align with the research of academics Meng et al. (2022), who shown that ISO can prevent the programmed cell death of brain cells in rats that have been harmed by Aβ25–35. Nevertheless, in recent years, the focus of ISO research has progressively transitioned to the study of tumors. According to literature, Shu et al. (2015) discovered that ISO can trigger programmed cell death in liver cancer cells by suppressing NF-κb. A study conducted by Law et al. (2016) discovered that ISO has the ability to trigger autophagic cell death in different types of cancer cells by activating AMPK. The study conducted by Li et al. (2022b) revealed that ISO triggers cell cycle arrest and death in cervical cancer cells by modulating the AKT/GSK3 pathway. Consequently, ISO is essential to studying and managing tumors (Cheng et al., 2021).

EMT stands for epithelial-mesenchymal transition, which is the process by which epithelial cells undergo transformation into mesenchymal cells, acquiring new properties. It has been established that in cancer, it plays a crucial role in the advancement of tumors, invasion, and the spread of cancer cells to other parts of the body. This mechanism makes cancer cells more aggressive (Yang and Weinberg, 2008). EMT is widely recognized as a crucial stage in the advancement and spread of GC. Its implications for the treatment and prognosis of GC are of great significance (Tian et al., 2020).

The TGF-β-Smad signaling system is a crucial cellular signaling route that significantly regulates diverse biological processes, including cell proliferation, differentiation, and migration (Hao et al., 2019). TGF-β interacts with its receptor TGFBR1 and exerts its biological effects via either the Smad signaling pathway or the non-Smad signaling route (Deng et al., 2024). During the advanced stage of malignancies, the TGF-β-Smad pathway triggers the process of EMT. It facilitates the spread of cancer cells by upregulating the expression of transcription factors, including Snai1 and Zeb1/2 (Hao et al., 2019). An excess of TGF-β can induce cell transition from an epithelial morphology to a more invasive spindle-shaped morphology (Xu et al., 2009). The observed morphological alteration is linked to the suppression of EMT under situations where TβR or Smad are lacking (Zhang et al., 2023). EMT induced by TGF-β also includes alterations in E-cadherin, N-cadherin, vimentin, and other proteins. These changes contribute to the enhanced invasiveness of tumors and their ability to metastasize (Saitoh, 2023). According to this, several outstanding prior studies (Wang et al., 2021; Shen et al., 2023; Du et al., 2021) have demonstrated the ability to suppress the growth and movement of cancer cells by targeting the TGF-β-Smad signaling pathway.

Considering the unique position of TGFBR1 in the TGF-β-Smad signaling pathway (Kwon et al., 2021; Yin et al., 2023), inhibition of TGFBR1 is considered an effective strategy to suppressing the TGF-β-Smad signaling pathway. Small-molecule TGFBR1 inhibitors, such as synthetic compounds SB-505124 (Hjelmeland et al., 2004), EW-7197 (Zeng et al., 2024), and SD-208 (Mohammad et al., 2011), and naturally occurring products like garcimultiflorone K (Huang et al., 2021), Galangin (Ru et al., 2021), and dihydromyricetin (Ye et al., 2019), have demonstrated potent anticancer activity in recent years. Clinical studies are currently evaluating EW-7197 among these inhibitor (Ahn et al., 2024).

Research has shown that elevated TGF-β levels in gastric cancer patients are associated with lymph node metastasis and a poor prognosis (Hu et al., 2014; Suda et al., 2009). High TGF-β levels have been observed in the gastric mucosa and stromal cells (Suzuki et al., 2018), resulting in poor clinical outcomes (Ma et al., 2013). In addition, the expression profile of gastric cancer is elevated in conjunction with an increase in the number of abnormally proliferating gastric epithelial cells, and TGFBR1 is also elevated (Kim et al., 2008). Moreover, TGFBR1 levels in adjacent tissues were significantly higher than in normal tissues (Zhang et al., 2021). Therefore, targeting TGF-β signaling pathway via the TGFBR1 may be an effective therapeutic strategy for the inhibition of gastric cancer metastasis.

Our initial investigations have demonstrated that ISO can suppress the growth of cervical cancer cells. During the initial bioinformatics investigation, we observed that ISO exerted its effects on the target gene TGFBR1 of GC. Furthermore, the molecular docking of ISO and TGFBR1 using Autodock revealed a strong binding activity. Thus, we postulated that ISO hinders the growth and movement of GC cells by controlling the TGF-β-Smad signaling pathway through its interaction with TGFBR1. Moreover, ISO suppressed the growth and movement of GC cells by stimulating the TGF-β signaling pathway. Furthermore, there was no significant disparity in the impact of siTGFBR1 and ISO therapy on GC cell proliferation, migration, and the TGF-β-Smad signaling pathway. To summarize, our findings demonstrate that ISO effectively suppresses the growth and movement of GC cells by specifically targeting TGFBR1 and modulating the TGF-β-Smad signaling pathway. This discovery establishes the foundation for identifying novel therapeutic targets for GC treatment.

## 2 Materials and methods

### 2.1 Cell

HGC27 was provided by the Institute of Drug Pharmacology, University of South China, while AGS was acquired from Cellverse Bioscience Technology Co., Ltd. (Shanghai, China). The cells were enriched with 10% fetal bovine serum (FBS; Biological Industries, Kibbutz Beit Haemek, Israel) and cultivated in RPMI1640 (1,640, Biological Industries, Kibbutz Beit Haemek, Israel) media containing 1% penicillin-streptomycin (Biological Industries, Kibbutz Beit Haemek, Israel). The cells were cultivated and maintained in a 37°C humidified incubator containing 5% CO_2_.

### 2.2 Experimental reagents

ISO was acquired from Shanghai Solarbio Bioscience & Technology Co., Ltd. (Shanghai, China). It was dissolved in dimethyl sulfoxide (DMSO; Mp Biomedicals Asia Pacific Pte Ltd., CA, United States). Transforming Growth Factor Beta-1 was acquired from novoprotein (Shanghai, China; cat. no. CA59). The TGFBR1 polyclonal antibody was obtained from proteintech (Wuhan, China; cat. no. 30117-1-AP). P-Smad2 (cat. no. 18338) was purchased from Cell Signaling Technology (CA, United States). The Smad2 (cat. no. R25742) antibody and the Smad4 (cat. no. R27290) antibody were purchased from ZEN-BIOSCIENCE (Chengdu, China). N-cadherin Antibody (cat. no. TA4039) was obtained from Abmart (Shanghai, China). The GAPDH (cat. no. AB0037), E-Cadherin (cat. no. CY1155) Antibody, Vimentin (cat. no. CY5134) Antibody, SNAI1 (cat. no. CY3066) Antibody, and Goat Anti-Rabbit IgG (H + L) FITC (cat. no. AB0121) were obtained from Abways (Shanghai, China). Goat anti-rabbit IgG (cat. no. A0208) was purchased from Beyotime (Shanghai, China).

### 2.3 Bioinformatics analysis

A schematic illustrating the workflow of the analysis is presented in graphical abstract. The Swiss Target Prediction website (http://www.swisstargetprediction.ch/) and the PharmMapper database (https://www.lilab-ecust.cn/pharmmapper/) identified 278 potential target genes for ISO. Subsequently, a total of 1404 GC target genes were chosen from the GeneCards database (https://www.genecards.org/), OMIM (https://omim.org/), and DisGeNET (https://www.disgenet.org/). The STRING database (http://string-db.org, Version 11.5) was utilized to build the protein-protein interaction (PPI) network, limited to the species “*Homo sapiens*”, with an interaction score greater than 0.4. The Cytoscape plug-ins Cytohubba and MCODE were employed to conduct a meticulous examination of topological parameters. GEPIA (http://gepia.cancer-pku.cn/) does Kaplan-Meier survival curve analysis and compares normal and malignant samples.

### 2.4 Molecular docking

The interaction between ISO and the protein was assessed by Molecular docking research. The 3D molecular structure of PPI was obtained in the SDF format from the PubChem database (https://pubchem.ncbi.nlm.nih.gov/). We utilize OpenBabel3.1.1 for the purpose of converting SDF files into molecules in PDB format. Additionally, the X-ray crystal structure data of TGFBR1 proteins (PDB ID: 6b8y) were acquired from the RCSB Protein Data Bank (PDB, http://www.rcsb.org/). Afterwards, we employed Autodock 1.5.6 for the process of semiflexible molecular docking. To perform a thorough examination of the outcomes, we utilized Pymol 2.5. Additionally, we employed visual analysis tools to present the two-dimensional (2D) structure diagram.

### 2.5 Cell proliferation assay

HGC27 and AGS cells, which had been cultured in flasks, were treated with 0.25% trypsin (VivaCell Biotechnology GmbH, Germany). The cells that were developing in a logarithmic manner were then transferred to 96-well plates, with each well containing 5 × 10^3^ cells. After the cells have attached, subject them to various concentrations of ISO (DMSO, 0, 5, 10, 20, 30, 40, 50, and 60 μM) and place them in a temperature-controlled incubator at a constant temperature of 37°C and 5% CO_2_ for 12, 24, and 36 h. Once finished, discard the medium and utilize a serum-free and Penicillin-Streptomycin-free medium to create a 10% CCK-8 (Yisheng Biotechnology Co., Ltd., Shanghai, China) solution. Subsequently, add 100 μL of the CCK-8 solution to each well. The 96-well plate was incubated at a temperature of 37°C for a duration of 30 min. Subsequently, it was transferred to a microplate reader called Epoch (BioTek Instruments, Inc. Vermont, United States) to quantify the absorbance at a wavelength of 450 nm, which was used to assess the vitality of the cells.

### 2.6 Colony formation assay

HGC27 and AGS cells, at a density of 800 cells per well, were placed in 6-well plates containing 2 mL of medium. Cells were stimulated with various doses of ISO (1, 2, 4, and 8 μM) and 0.1% DMSO for 24 h. Once a colony of cells surpasses 50 cells, dispose of the medium, rinse it three times with physiological saline, immobilize it with 4% paraformaldehyde (Biosharp, China) for 30 min, eliminate the paraformaldehyde, rinse it three times with physiological saline. The 0.1% crystal violet staining solution (Solarbio, China) was further stained for an additional 20 min. The staining solution was rinsed with micro-flow water, transferred to a fluorescence microscope (BioTeck Instruments, Inc. Vermont, United States), and captured in a bright field image.

### 2.7 Transwell assay

The evaluation of cellular invasion was performed using Transwell assays. To summarise, the upper section of the transwell device was coated with a diluted Matrigel solution (Corning, China) at a ratio of 1:8. HGC27 and AGS cells were diluted in serum-free 1,640 medium and added to the upper chambers at a density of 1.0 × 10^5^ cells in 200 µL of cell suspension. The lower compartments were filled with medium containing 10% FBS, which served as a chemoattractant to enhance cell penetration. Subsequently, the chambers were subjected to ISO concentrations of 10 μM and placed in a humid environment at a temperature of 37°C with 5% CO_2_ for 24 h. The non-invasive cells on the membrane’s top layer were gently removed using a cotton swab. In contrast, the cells introduced into the lower chamber were immobile, 0.1% crystal violet staining solution stained 20 min, and observed under a microscope (Leica DM IL LED, Germany). The number of invading cells in each group was quantified using ImageJ software.

### 2.8 Wound healing assay

The assessment of cellular migration was performed using the Wound Healing Assay. In conclusion, HGC27 and AGS cells were cultivated in 6-well plates until they established a cohesive monolayer. Afterwards, a 100-μL pipette was used to create an apparent scratch wound extending across each well’s center. Subsequently, the floating cells were eliminated, and a fresh serum-free 1,640 medium containing 10 μM ISO was introduced into each well. Subsequently, the 6-well plates were placed in a CO_2_ incubator to promote cell migration. Photographic documentation of each wound was conducted at regular 0 h, 10 h and 24 intervals, and the motion was quantified using ImageJ software. Migration (%) = (initial scratch area-final scratch area)/(initial scratch area) × 100%

### 2.9 Western blot analysis

The cells cultured in the six-well plate were washed thrice with physiological saline and placed in cold storage. Each treatment group was subjected to lysis for 10 min by adding an equivalent volume of RIPA solution (Beyotime Biotechnology, Shanghai, China) with PMSF and phosphatase inhibitors. Use the BCA kit (Epizyme Biotech, Shanghai, China) to determine protein content. Use lysis buffer to ensure equal protein content in each group. Finally, add loading buffer and heat the sample in a metal bath at 100°C for 10 min. The material is placed onto a gel and then partitioned. Subsequently, it is transferred to a polyvinylidene fluoride (PVDF) membrane employing a current of 200 mA for 60 min. Following TBST cleaning of the PVDF membrane, the membrane was blocked for 15 min using Protein Free Rapid Blocking Buffer (Epizyme Biotech; cat. no. PS108). After washing with TBST, place the PVDF membrane in direct contact with the designated concentration of primary antibody (1:500–1:2000) and incubate it at 4°C overnight. The PVDF membrane was cleaned and then incubated with HRP goat anti-rabbit secondary antibody for 1 h at room temperature with agitation on a shaker after being removed and left overnight. Following three washing cycles with TBST, the sample underwent additional processing in a developing apparatus. Before the development process, each film was submerged in the developer solution for 2 min to guarantee a comprehensive and meticulous amalgamation of the developer and the film. ImageJ analyzed the developmental observations for the grayscale intensity.

### 2.10 Small interfering RNA (siRNA) transfection

Dilute the siRNA concentration to 20 µm by adding DEPC water. Firstly, the cell solution is added to the cell culture plate. The cell confluence before transfection varies between 60% and 80%. Complex configuration: Combine the nucleic acid with Invigentech INVI DNA RNA transfection reagent (Invigentech, CA, United States) in equal amounts, gently mix by pipetting 10–15 times, and allow it to sit at room temperature for 10–15 min. Add the complex substance to the cell culture plate and gently mix it. Afterwards, the plate is transferred to an incubator for growth, and the nutrient solution is usually changed 24 h after transfection. The siRNA sequence is shown in Table 1.

**TABLE 1:** The sequences of siRNAs.

siRNA- TGFBR1		
si1	Sense	5´-GCC​UUA​UUA​UGA​UCU​UGU​ATT-3´
Antisense		5´-UAC​AAG​AUC​AUA​AUA​AGG​CTT-3´
si2	Sense	5´-GCA​ACU​CAG​UCA​ACA​GGA​ATT-3´
Antisense		5´-UUC​CUG​UUG​ACU​GAG​UUG​CTT-3´
si3	Sense	5´-CGU​GCU​GAC​AUC​UAU​GCA​ATT-3´
Antisense		5´-UUG​CAU​AGA​UGU​CAG​CAC​GTT-3´

### 2.11 Immunofluorescence analysis

Place the coverslip into a six-well plate and distribute the cells evenly until they achieve a growth rate of 60% before administering the medication. Following the procedure, fix the slide by applying a 4% paraformaldehyde solution for 15 min. Afterwards, they increase the permeability of the cells by subjecting them to a 0.5% Triton-X100 (Solarbio, Beijing, China) solution for 20 min. This step does not apply to membrane proteins such as E-cadherin and N-cadherin. After completing the permeabilization process, wash with TBST and then expose to the designated concentration of primary antibody (1:200) at 4°C overnight. After incubating the sample with the primary antibody, wash it and then incubate it in a 37°C incubator for 1 h with a fluorescent secondary antibody (1:200) in a light-free environment. After removing the excess fluorescent secondary antibody, slides coated with DAPI containing anti-fluorescence quenching (Solarbio; Cat. No. S2110). Finally, photographs was taken using a fluorescence microscope (BioTeck Instruments, Inc. Vermont, United States).

### 2.12 Statistical analysis

The studies were performed in three distinct iterations, and the data is presented as the average value with the standard deviation (SD) indicated. The analysis was conducted using GraphPad Prism 9.0. The independent-sample t-test was used to compare two data sets, whereas Tukey’s *post hoc* test was used to analyze numerous data sets. The significance level was set at a p-value < 0.05. The experiment was performed three times in a manner that was not impacted by earlier iterations.

## 3 Results

### 3.1 ISO target protein TGFBR1 is highly expressed in GC cells

**FIGURE 1:**
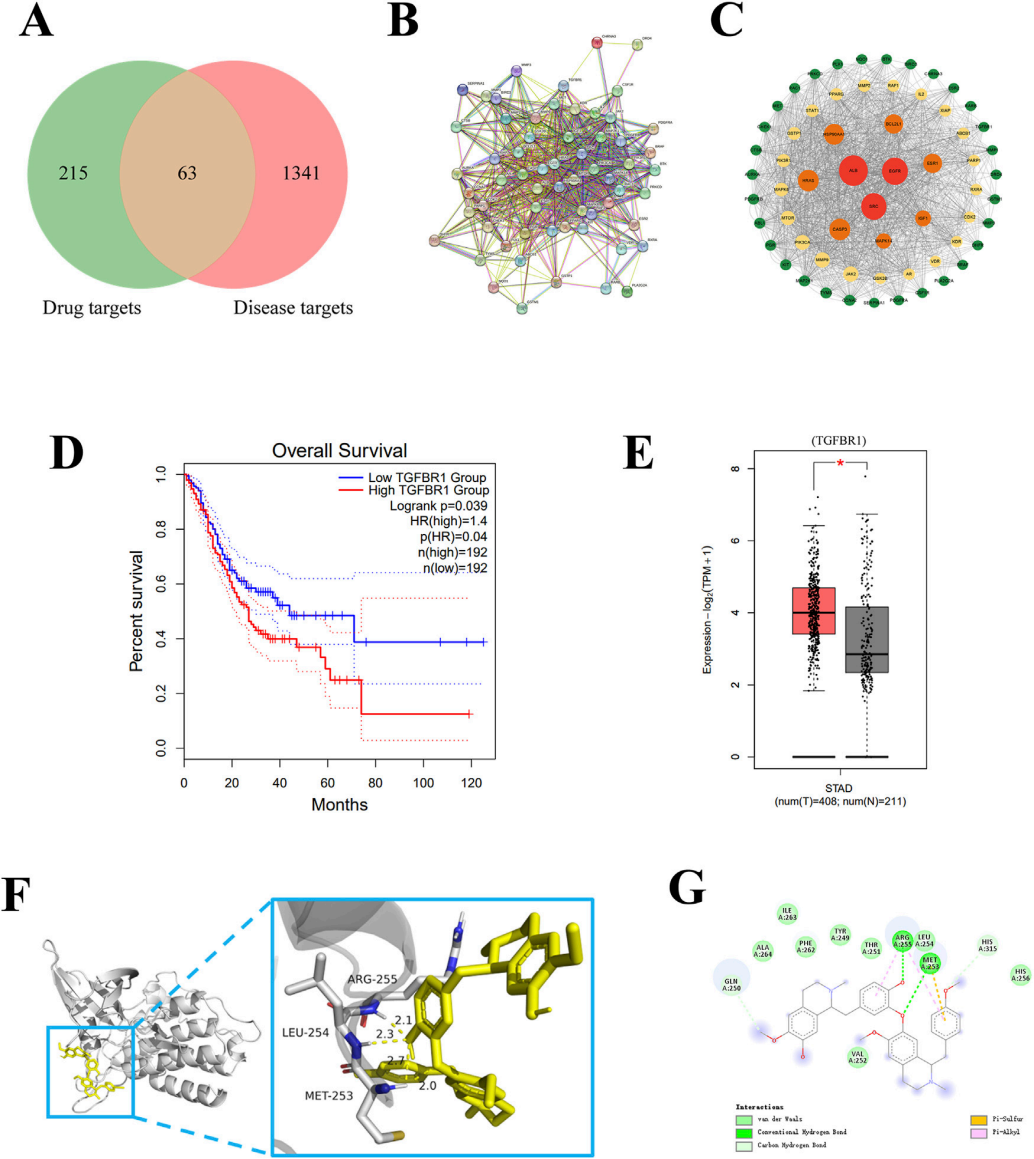
The target of ISO in GC was analyzed using bioinformatics. **(A)** Diagram illustrating the overlapping areas between the ISO target and GC co-target using a Venn diagram. **(B, C)** The diagram depicts the relationship between ISO and GC in a PPI network. **(D)** The Kaplan-Meier survival curves of TGFBR1 in GC were analyzed. Genes with no association with TGFBR1 are represented in blue, genes with a negative correlation are represented in red, and genes with a positive correlation are depicted in green. **(E)** Differential expression analysis of TGFBR1 in cancer and para-cancerous tissue, red represents cancer tissue, and gray represents para-cancerous tissue. **(F)** The molecular docking data for TGFBR1 indicate a binding energy of −6.64 kcal/mol and the potential formation of at least two hydrogen bonds with TGFBR1. **(G)** 2D visualization of molecular docking results using online tools.

**FIGURE 2:**
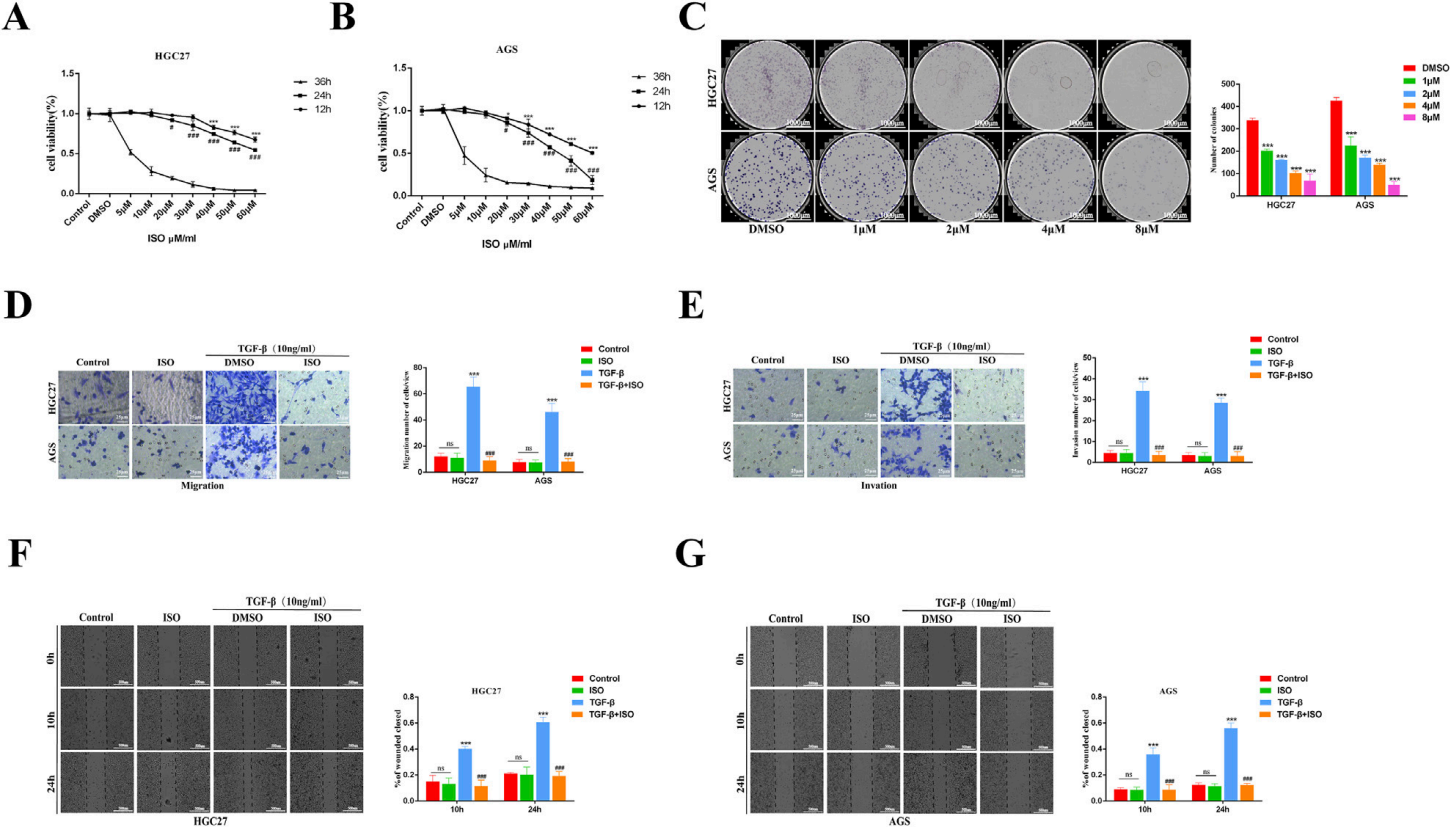
The impact of ISO on the growth, movement, and infiltration of stomach cancer cells. **(A, B)** CCK-8 is used to assess the cell survival of HGC27 and AGS GC cells after treatment with ISO at various concentrations and time gradients. **(C)** After 7 days of treatment with ISO (DMSO, 1, 2, 4, 8 μM), colony development and statistical results were observed. **(D, E)** Following a 2 h pretreatment with 10 μM ISO, TGF-β stimulation for 24 h, the rate of cell penetration was measured using transwell. The statistics show the number of HGC27 and AGS cells that invade and migrate within 24 h. **(F, G)** Utilizing a quantity of 10 μM of ISO Following a pretreatment period of 2 h, TGF-β was administered for 10 h and 24 h using a wound healing test to assess the impact of ISO on the migratory capacity of HGC27 and AGS cells. The statistical results are shown as the ratio of cell migration compared to the initial migration area at 0 h. Results are expressed as means ± SEM, n = 3. * *P* < 0.05, *** *P* < 0.001 when compared to DMSO group. # *P* < 0.05, ### *P* < 0.001 when compared to the Control group (not A, B).

To identify the target of ISO on GC cells, we utilized Swiss Target Prediction (http://www.swisstargetprediction.ch/) and PharmMapper database (https://www.lilab-ecust.cn/pharmmapper/) to choose 278 probable target genes of ISO. Afterwards, a collective sum of 1,404 genes associated with GC (gastric cancer) were obtained from the GeneCards (https://www.genecards.org/) database, OMIM (https://omim.org/), and DisGeNET (https://www.disgenet.org/) individually. When ISO-related and GC-related targets were analyzed using a Venn diagram, it was found that 63 target genes of ISO act on GC cells (Figure 1A). Subsequently, 63 genes were entered into the STRING database to create a PPI network (Figure 1B). The software Cytoscape 3.9.1 was employed to develop a comprehensive target network in GC. The composite target network comprised 63 nodes and 800 edges (Figure 1C). We utilized the GEPIA website to construct the Kaplan-Meier survival curve for 63 genes in the PPI network. We employed the log-rank test to evaluate the survival rates between groups with high and low expression, with a significance threshold of P < 0.05. As a result, we identified 25 genes that exhibited significant variations in survival (Figure 1D and Supplementary Figure S3). Next, we analyzed the disparities between regular and neighbouring cancer tissues about the 25 genes that showed variations in survival rates. We observed differential expression of four genes, ALB, KIT, PDGFRB, and TGFBR1, in regular and neighbouring cancer tissues (Figure 1E and Supplementary Figure S4). Ultimately, we employed autodock software to perform molecular docking analysis (Figure 1F) and 2D visualization analysis (Figure 1G) on these four points. Our findings indicate that ISO exhibits superior binding activity with TGFBR1 (Binding energy = −6.64 kcal/mol) and can form at least two hydrogen bonds with TGFBR1.

### 3.2 ISO inhibits the proliferation, migration and invasion of GC cells

The impact of ISO on the proliferation of HGC27 and AGS cells was assessed using the CCK-8 assay. The findings demonstrated that ISO exerted a suppressive effect on cell growth, with the degree of inhibition dependent on the dosage and duration of exposure (Figures 2A, B). The suppressive impact of ISO on the development of HGC27 and AGS cells was additionally assessed by a colony formation experiment. According to the results, there was a considerable decrease in the number of colonies formed by HGC27 and AGS cells as the ISO concentration increased. The statistical analysis demonstrated that ISO suppressed the formation of colonies by HGC27 and AGS cells in a manner dependent on the ISO concentration (Figure 2C). The impact of ISO on the migration and invasion of GC cells was assessed using Transwell and scratch tests. The Transwell experiment demonstrated that applying ISO (10 μM) effectively decreased the cellular infiltration rate caused by TGF-β (Figures 2D, E). The results of the scratch experiment further demonstrate that treatment with ISO (10 μM) can effectively decrease the cell migration rate induced by TGF-β. The ISO compound exhibits a cell migration suppression rate of over 50% compared to the control group treated with DMSO. This inhibition is significantly higher than the impact of ISO (10 μM) on cell viability. ISO demonstrates an inhibitory effect on the migratory activity of GC cells (Figures 2F, G).

### 3.3 ISO inhibits TGF-β-induced tumor EMT

**FIGURE 3:**
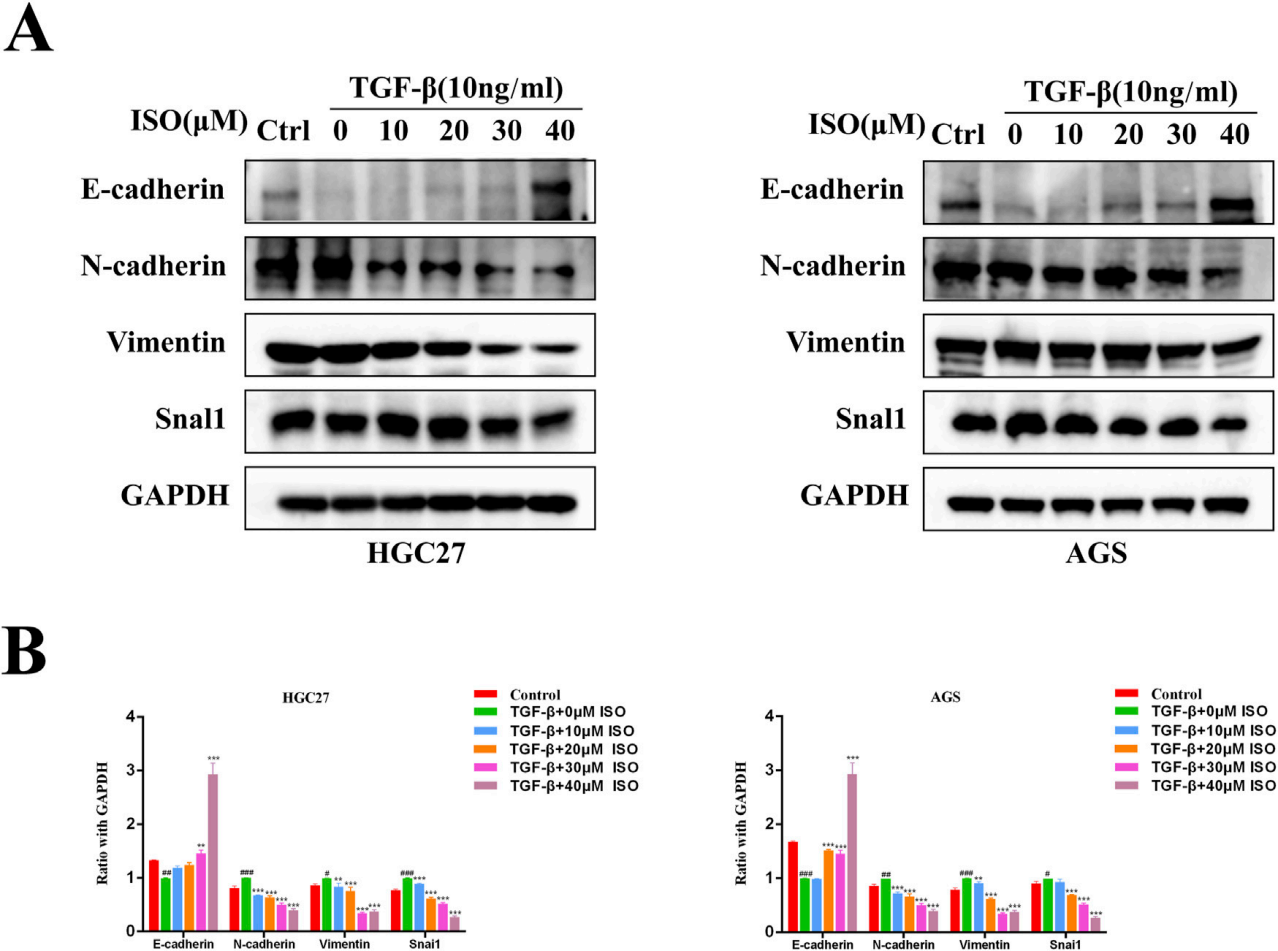
Western blotting is used to measure the impact of ISO on the EMT of gastric cancer. **(A)** Pretreatment with different concentrations of ISO (0, 10, 20, 30, 40 μM) for 2 h, followed by TGF-β induction for 24 h, and Western blotting to detect the levels of E-cadherin, N-cadherin, Vimentin and Snai1 in HGC27 cells and AGS cells. **(B)** Statistical data on the relative protein expression in HGC27 and AGS cells. Results are expressed as means ± SEM, n = 3. * *P* < 0.05, ** *P* < 0.01 and *** *P* < 0.00, compared to the DMSO group. # *P* < 0.05, ## *P* < 0.01, ### *P* < 0.001 when compared to Control group.

**FIGURE 4:**
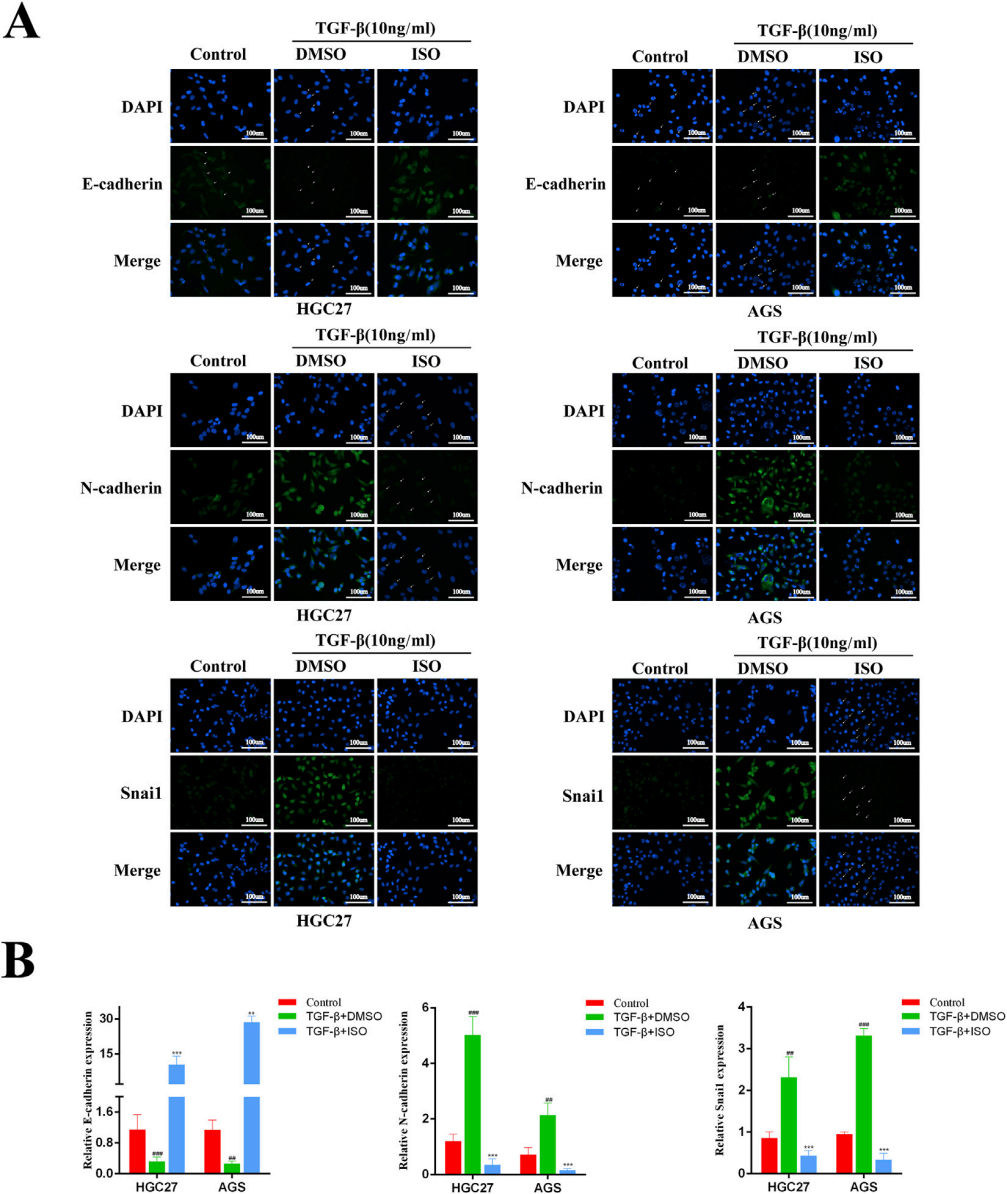
Immunofluorescence detection the impact of ISO on the EMT process in gastric cancer. **(A)** Immunofluorescence detection of E-cadherin, N-cadherin, Vimentin and Snai1 levels in HGC27 cells and AGS cells after two h pretreatment with 10 μM ISO and TGF-β induction for 24 h. **(B)** Quantitative assessment of the intensity of fluorescence. Results are expressed as means ± SEM, n = 3.** *P* < 0.01 and *** *P* < 0.001, when compared to DMSO group.## *P* < 0.01, ### *P* < 0.001 when compared to Control group.

To investigate the impact of ISO on the EMT signalling pathway triggered by TGF-β, we employed Western blotting and immunofluorescence techniques to examine the influence of ISO on tumour EMT generated by TGF-β. The Western blotting study demonstrated that ISO therapy effectively reversed the TGF-β-induced downregulation of E-cadherin expression and counteracted the TGF-β-induced upregulation of N-cadherin, vimentin, and Snai1 expression, as compared to the DMSO control group (Figure 3A). Furthermore, the statistical analysis revealed significant differences (Figure 3B). Moreover, we employed immunofluorescence to identify the presence of E-cadherin, N-cadherin, and Snai1 proteins. Consistent with the findings from Western blotting analysis, the activation of TGF-β resulted in a decrease in the brightness of E-cadherin (green) fluorescence while increasing the brightness of N-cadherin (green) and Snai1 (green) fluorescence. After treating the cells with ISO, the downregulation of E-cadherin expression resulting from TGF-β was reversed, along with the upregulation of N-cadherin and Snai1 expression caused by TGF-β (Figures 4A, B). This indicates that ISO has the potential to reverse the TGF-induced EMT of GC cell lines.

### 3.4 ISO inhibits the activation of the TGF-β-Smad pathway

**FIGURE 5:**
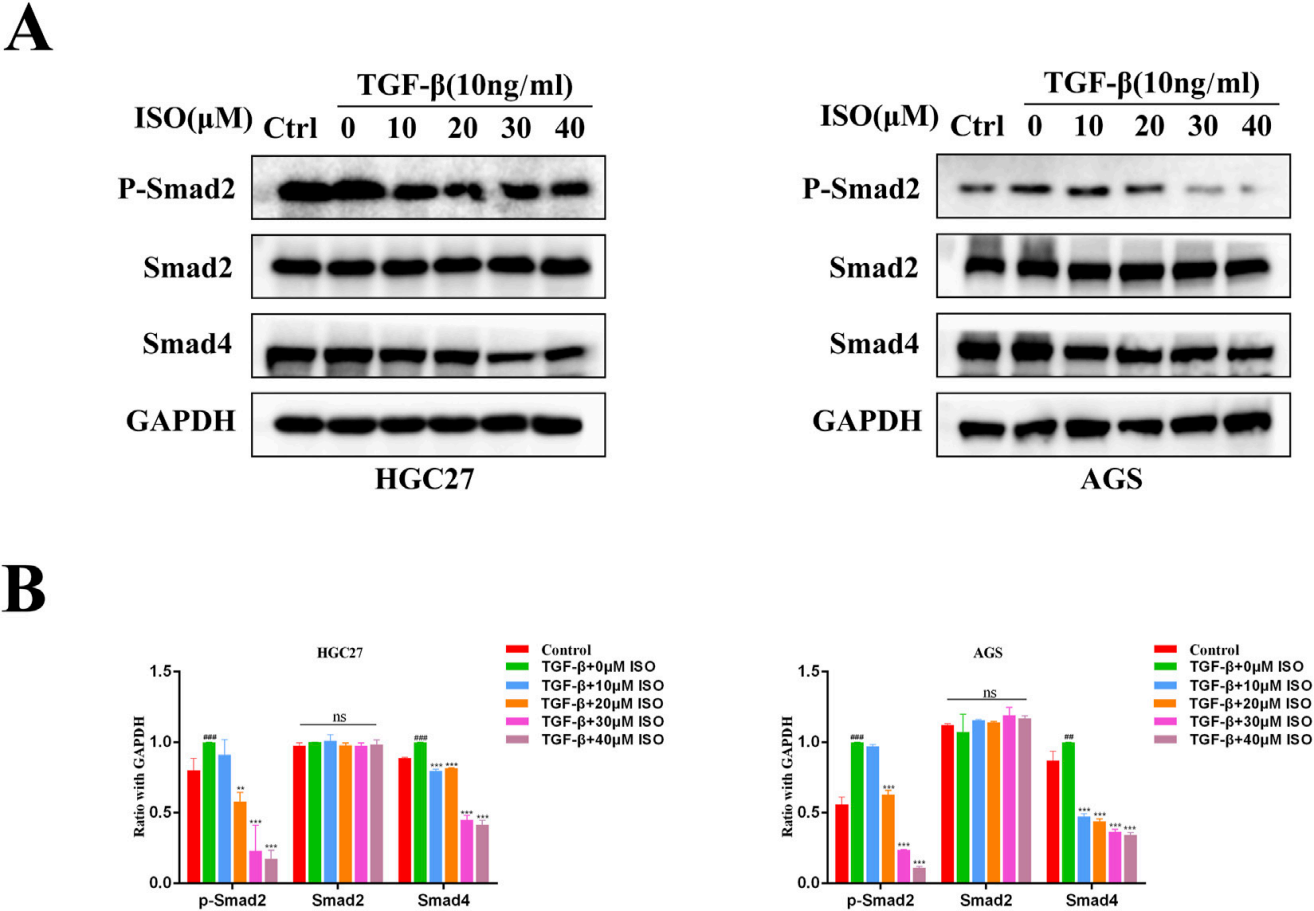
Western blotting assesses the impact of ISO on the proteins involved in the TGF-β-Smad pathway. **(A)** Pretreatment with different concentrations of ISO (0, 10, 20, 30, 40 μM) for 2 h, followed by TGF-β induction for 24 h, and Western blotting to detect the levels of p-Smad2, Smad2, and Smad4in HGC27 cells and AGS cells. **(B)** The HGC27 and statistical data pertain to the relative protein expression in AGS cells. Results are expressed as means ± SEM, n = 3. ** *P* < 0.01, *** *P* < 0.001and ns, not significant (*P* > 0.05) when compared to the DMSO group. # *P* < 0.05, ## *P* < 0.01, ### *P* < 0.001 when compared to Control group.

**FIGURE 6:**
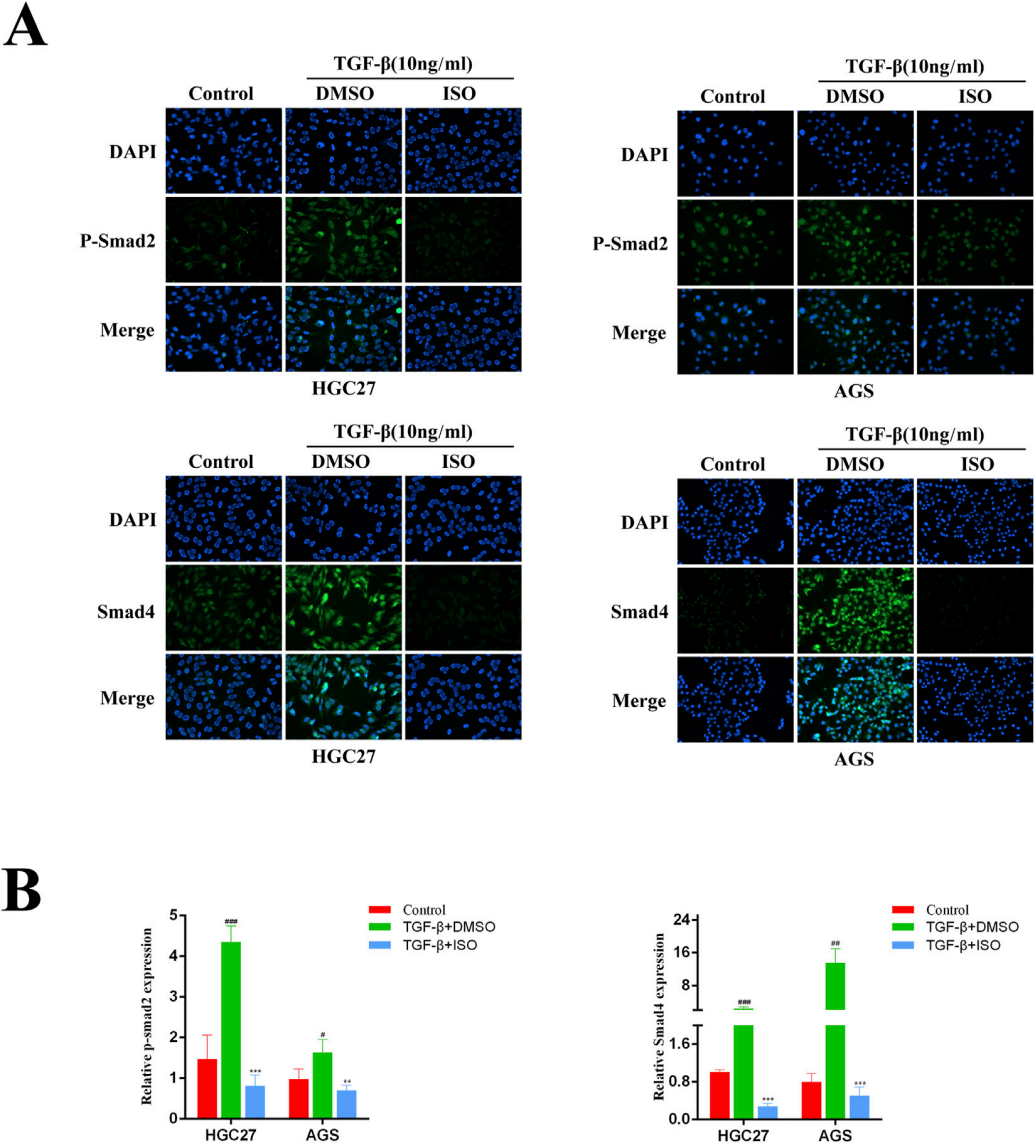
Immunofluorescence was used to evaluate the influence of ISO on the proteins relevant to the TGF-β-Smad pathway. **(A)** Immunofluorescence detection of p-Smad2, Smad2, and Smad4 levels in HGC27 and AGS cells after two h pretreatment with 10 μM ISO for two h and TGF-β induction for 24 h. **(B)** Immunofluorescence was used to detect the impact of ISO on the proteins involved in the TGF-β-Smad pathway. Results are expressed as means ± SEM, n = 3. ** *P* < 0.01 and *** *P* < 0.001, compared to the DMSO group. # *P* < 0.05, ## *P* < 0.01 and ### *P* < 0.001 when compared to Control group.

To investigate the impact of ISO on the TGF-β-Smad pathway, we employed Western blotting and immunofluorescence techniques to examine the influence of ISO on the crucial proteins p-Smad2, Smad2, and Smad4 within the TGF-β-Smad pathway. The Western blotting study demonstrated that ISO therapy effectively counteracted the TGF-β-induced upregulation of p-Smad2 and Smad4 expression compared to the DMSO control group (Figure 5A). By doing statistical analysis, it was shown that there were significant statistical disparities (Figure 5B). Furthermore, we employed immunofluorescence to identify the presence of p-Smad2 and Smad4 proteins. Consistent with the findings from Western blotting analysis, the fluorescence intensity of p-Smad2 (green) and Smad4 proteins (green) increased with TGF-β stimulation. However, ISO treatment reversed the TGF-β-induced increases in p-Smad2 and Smad4 (Figures 6A, B). In addition, we also detected the expression of P-Smad2 and Smad4 1 h after TGF-β induction by Western blotting and immunofluorescence techniques (Supplementary Figure S1; Supplementary Figure S2). We found that the overexpression of p-Smad2 and Smad4 induced by 1 h TGF-β induction was also inhibited by ISO. This suggests that ISO can inhibit the activation of the TGF-β-Smad pathway.

### 3.5 Knocking down TGFBR1 counteracts the inhibitory effects of ISO on GC cell migration and invasion

**FIGURE 7:**
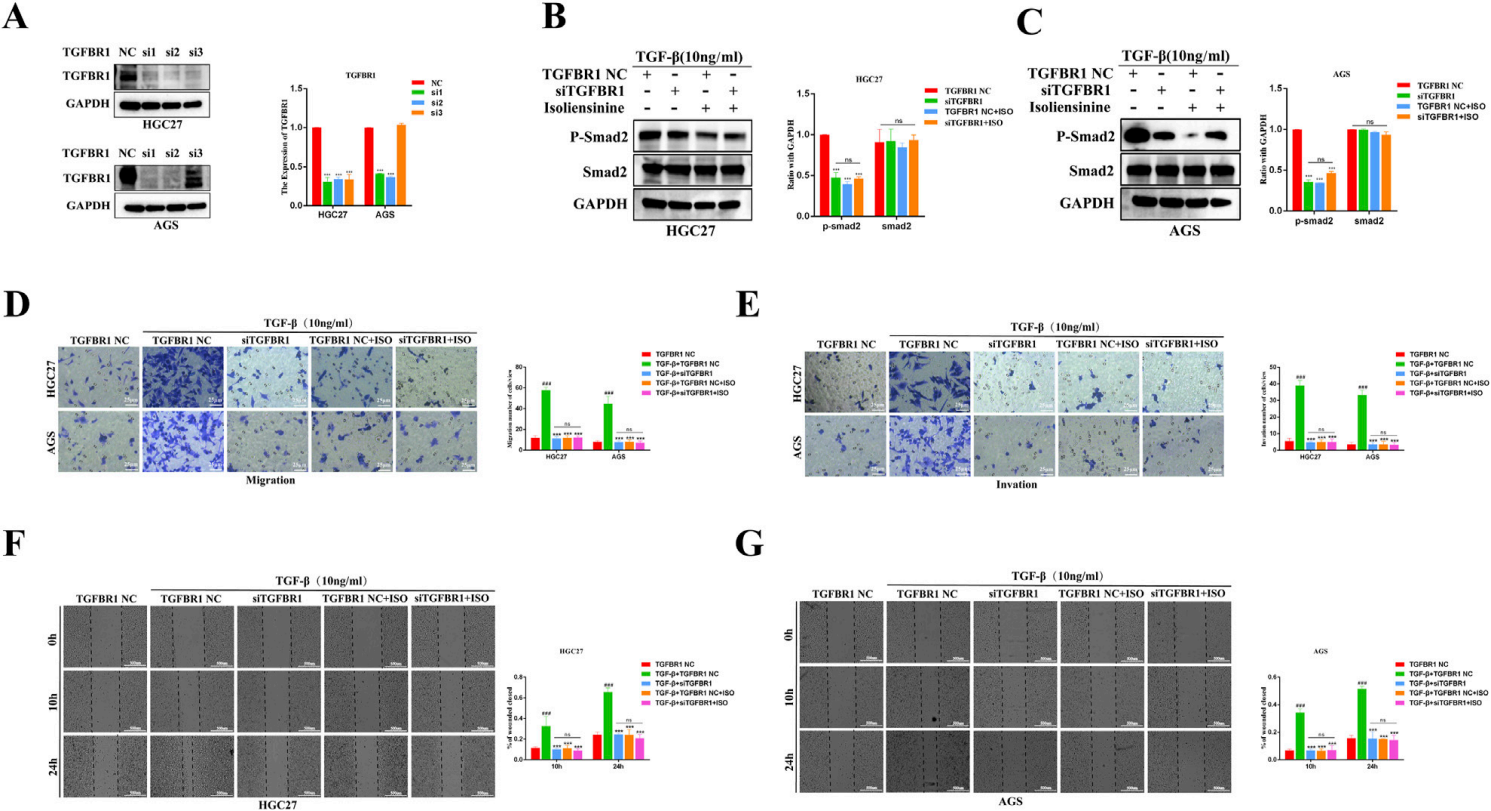
Following siTGFBR1, the impact of ISO on the TGF-β-Smad pathway and the migration and invasion of gastric cancer cells was assessed. **(A)** Western blotting to detect the knockout efficiency of TGFBR1 by three siRNAs. Statistical data on the relative protein expression of TGFBR1 after siRNA treatment. **(B, C)** Western blotting was performed to detect the presence of P-Smad2 and Smad2 proteins following treatment with siTGFBR1 and ISO 10 μM. The symbol “+” indicates treatment, whereas the symbol “-“ indicates no treatment. Statistical data on the expression of P-Smad2 and Smad2 proteins following various treatments. **(D, E)** The number of transwell migration and invasion cells following TGF-induction under various treatment conditions. The statistics show the number of HGC27 and AGS cells that invade and migrate within 24 h. **(F, G)** The effect of ISO on the migratory ability of HGC27 and AGS cells 10 h and 24 h after TGF-β stimulation was examined using a wound healing assay under different treatment settings. The statistical data are shown as cells’ migration area coverage rate relative to the initial time point (0 h). Results are expressed as means ± SEM, n = 3. *** *P* < 0.001and ns, not significant (*P* > 0.05) when compared to DMSO group. ### *P* < 0.001 when compared to the Control group.

To further verify the inhibitory effect of ISO on the TGF-β-Smad signaling pathway and cell metastasis, siRNA was used to transfect HGC27 and AGS cells to downregulate the expression of TGFBR1. Western Blot results showed that si2 had a better transfection effect on the two cell lines (Figure 7A). In the experiment, we used si2 to conduct subsequent knockdown experiments. The results showed that the knockdown of TGFBR1 significantly reduced the expression of p-Smad2. However, co-treatment of ISO (40 μM) and TGFBR1 failed to reduce the expression of p-Smad2 further, and there was no significant change in the expression of Smad2 in each group (Figures 7B, C). Next, the effects of ISO and TGFBR1 knockdown on tumor cell migration and invasion were determined by transwell assay (Figures 7D, E) and scratch assay (Figures 7F, G). We found that TGFBR1 knockdown inhibited TGF-β-induced GC cell migration and invasion, similar to the inhibitory effect of ISO (10 μM). However, the effect of combined application of siTGFBR1 and ISO (10 μM) on cell migration and invasion ability was not significantly different from that in the siTGFBR1 group, suggesting that siTGFBR1 gene knockdown can offset the therapeutic effect of ISO (10 μM). The above results further indicate that TGFBR1 promotes the metastasis of tumor cells, and ISO inhibits the migration and invasion of tumor cells by inhibiting TGFBR1.

## 4 Discussion

The TGF-β-Smad pathway plays a crucial role in promoting tumor development by inducing EMT, facilitating interactions between tumors and the surrounding stroma, and aiding in the evasion of immune recognition by malignancies (Gough et al., 2021). The signaling has the potential to be targeted therapeutically for a range of disorders, such as organ fibrosis and malignancies (Ghafouri-Fard et al., 2024; Wu et al., 2021b). A number of therapeutic strategies have been created to target the TGF-β-Smad pathway, including antisense nucleic acids, ligand traps, monoclonal antibodies, and small molecule inhibitors. Some of these techniques have progressed to the clinical trial phase (Syed, 2016). TGFBR1 inhibitors play a crucial role in developing medications targeting the TGF-β-Smad signaling pathway, one of the strategies used to treat malignancies (Fan et al., 2023; Cao et al., 2024).

It is understood that drugs for the treatment of GC have been reported to have pronounced side effects, and traditional Chinese medicine, as a natural product, has the characteristics of minor side effects. Many studies have shown that many traditional Chinese medicines have a therapeutic effect on GC, such as berberine, Dihydroisotanshinone I, etc. (Zhang et al., 2020; Wu et al., 2021a). Research has confirmed that ISO can mediate autophagy in lung cancer cells by affecting mTOR (Ren et al., 2023) and activate mitochondria through PI3K/PAKT/mTOR to mediate apoptosis in rectal cancer cells (Manogaran et al., 2019); in addition, it can also induce apoptosis in breast cancer cells (Zhang et al., 2015). Inhibiting NF-κB induces apoptosis in liver cancer cells (Shu et al., 2015). Our group’s previous research has shown that it inhibits the cycle and apoptosis of cervical cancer cells through the AKT/GSK3α signaling pathway (Li et al., 2022b). However, so far, the effect of ISO on GC cells and its anti-tumor mechanism is still unknown.

To assess the impact of ISO on GC cells, we employed bioinformatics techniques to analyze the PPI network between ISO and target genes associated with GC. We have identified 63 candidate target genes of ISO on GC cells. Subsequently, 63 genes were examined to identify variations in expression between GC tissues and surrounding tissues. As a result, 25 genes were identified as having significant differential expression. Finally, we conducted KM survival analysis on a set of 25 genes that showed significant differential expression and 4 genes (ALB, KIT, PDGFRB, and TGFBR1) that exhibited survival differences. Among these genes, TGFBR1 has been previously studied in the context of GC targeting. To investigate its potential interaction further, we employed autodock molecular docking software to dock ISO and TGFBR1. The docking activity of this interaction was determined to be −6.64 (kcal/mol), indicating a strong affinity between ISO and TGFBR1. A total of two hydrogen bonds were established. Subsequently, a 2D demonstration of the molecular docking results was performed using visualization software. It has been discovered that ISO can attach itself to the specific amino acid residues (ARG255, LEU254, and MET253) located in the active pocket of TGFBR1. Therefore, ISO may impact the proliferation and migration of GC cells by explicitly targeting TGFBR1.

TGF-β is the most common stimulator for studying EMT in diverse cellular systems. The biochemical changes that occur when epithelial cell biomarkers decline and mesenchymal cell biomarkers increase during epithelial cell to mesenchymal cell transformation confirm that EMT has occurred (Neuzillet et al., 2015). The results of our investigation shown that ISO effectively suppressed cell migration and invasion induced by TGF-β at low and non-cytotoxic doses. Additionally, ISO had a significant inhibitory effect on tumor cell growth at relatively high concentrations. Moreover, in AGS cells and HGC27 cells, TGF-β treatment induced an increase in Snai1 and N-cadherin proteins and a decrease in E-cadherin expression by Western blotting and immunofluorescence assays, suggesting that EMT occurred. Our findings indicate that these changes were inhibited and reversed following ISO pretreatment, which suggests that ISO inhibited TGF-β-induced EMT. In addition, similar to our findings, the natural product Toosendanin (Luo et al., 2018) inhibits the TGF-β-Smad signaling pathway, reduces snail expression at low concentrations, inhibits cell migration and invasion, and slows down cell proliferation at high concentrations, which may be due to the multifunctional properties of the TGF-β-Smad signaling pathway in regulating cell proliferation (Cheng et al., 2015; Datto et al., 1995), apoptosis (Tobin et al., 2001), migration, and invasion. Furthermore, to evaluate the impact of ISO on TGFBR1, we used Western blot and immunofluorescence to detect the expression of the TGF-β-Smad pathway protein. Our findings revealed that ISO treatment reversed the expression of TGF-β-induced p-Smad2 and Smad4, indicating that ISO treatment inhibited TGF-β-induced activation of the TGF-β-Smad pathway. In addition, since the TGF-β-induced Smad phosphorylation was higher after TGF-β stimulation for 0.5–1 h (Peterson et al., 2022), in order to avoid the indirect effect of the TGF-β-Smad signaling pathway, we detected the TGF-β-Smad pathway with TGF-β stimulation for 1 h and found that ISO at this time point could reverse the activation of the TGF-β-Smad pathway induced by TGF-β. This indicates that ISO can indeed affect the TGF-β-Smad pathway. More importantly, We found that siTGFBR1 inhibited TGF-β-induced tumor cell migration and invasion, similar to the inhibition of ISO. In addition, the effects of ISO and siTGFBR1 on cell invasion and migration were similar to those of siTGFBR1, suggesting that the inhibition of ISO was offset by TGFBR1 knockdown. These results further suggest that ISO can inhibit the migration and invasion of gastric cancer cells by directly inhibiting TGFBR1.

Furthermore, our cellular tests revealed that ISO therapy effectively suppressed the development and movement of gastric cancer cells. This inhibition was accompanied by a decrease in the expression of proteins associated with the EMT phenotype and the TGF-β-Smad pathway. Significantly, siTGFBR1 effectively reversed the inhibitory impact of ISO on GC cell motility and the TGF-β-Smad signaling pathway.

## 5 Conclusion

**FIGURE 8:**
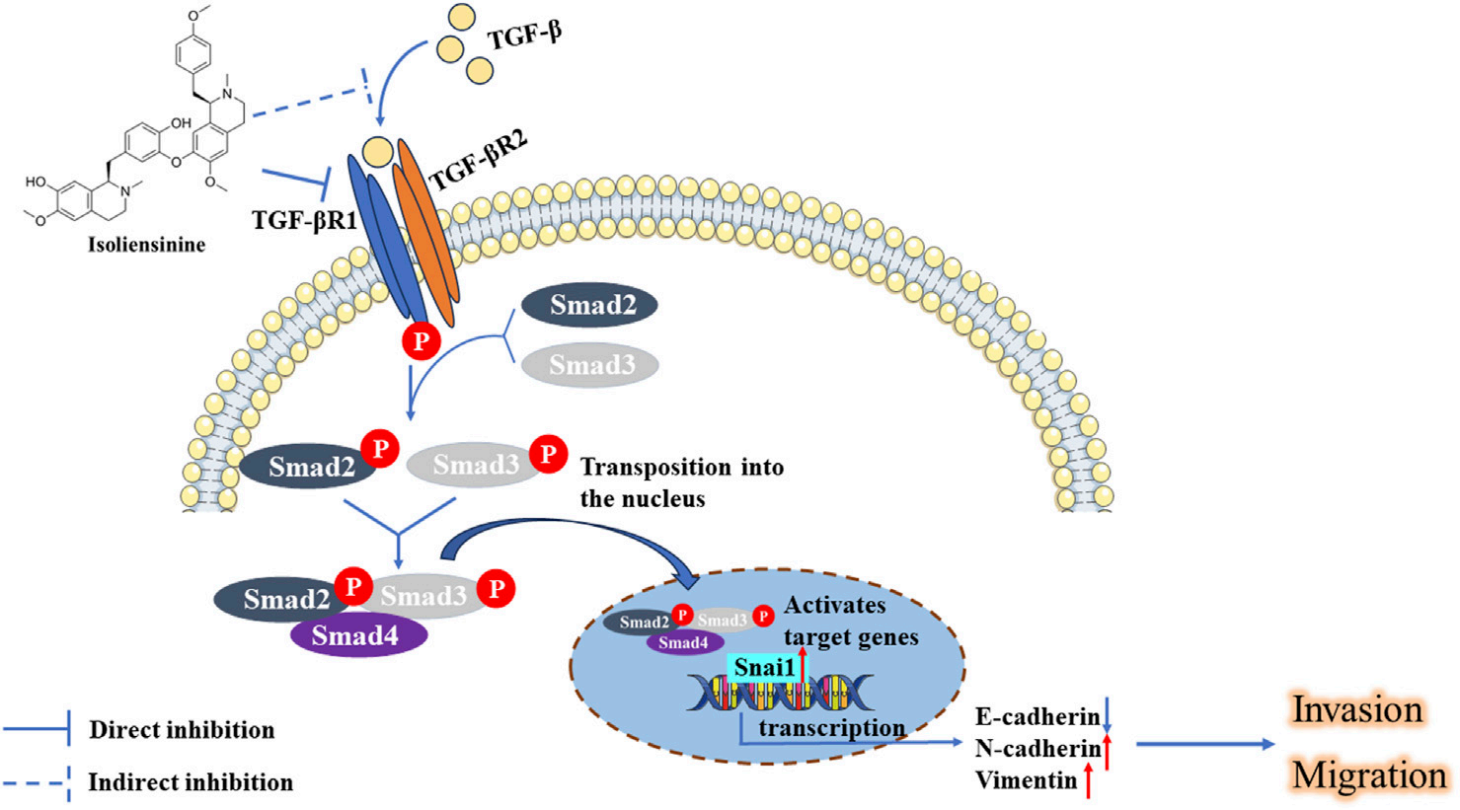
ISO inhibited the growth and movement of triple-negative breast cancer cells by targeting TGFBR1 to regulate the TGF-β-Smad signaling pathways.

In this study, we systematically studied the mechanism by which ISO inhibits the TGF-β-Smad signaling pathway to inhibit the migration and invasion of GC cells (Figure 8). We first found that ISO inhibited the proliferation and migration of GC cells, which further inhibited the EMT of GC cells. Furthermore, ISO targets TGFBR1 and ultimately inhibits the TGF-β-Smad signaling pathway. Our study developed an inhibitor targeting TGFBR1, and inhibiting the TGF-β-Smad pathway by targeting TGFBR1 via ISO may provide a feasible strategy for treating GC.

## Data Availability

The original contributions presented in the study are included in the article/Supplementary Material, further inquiries can be directed to the corresponding author.
