# Supplementary material for: Isoliensinine suppressed gastric cancer cell proliferation and migration by targeting TGFBR1 to regulate TGF-β-smad signaling pathways
Source: Front Pharmacol. 2024 Sep 19;15:1438161. doi: 10.3389/fphar.2024.1438161 (PMC11446791; doi:10.3389/fphar.2024.1438161)
Supplement: Supplementary file 1 [file DataSheet1.docx]

Supplementary Material

Original western blots description: All protein bands are repeated three times (marked as red 1, 2, 3 in the picture). Each protein band contains the grayscale, brightfield, and merge images of the protein and the corresponding internal reference (They are marked as PUB, Maker, PUB+Maker in the figure).

# Original western blots for Figure 4(E-Cadherin)
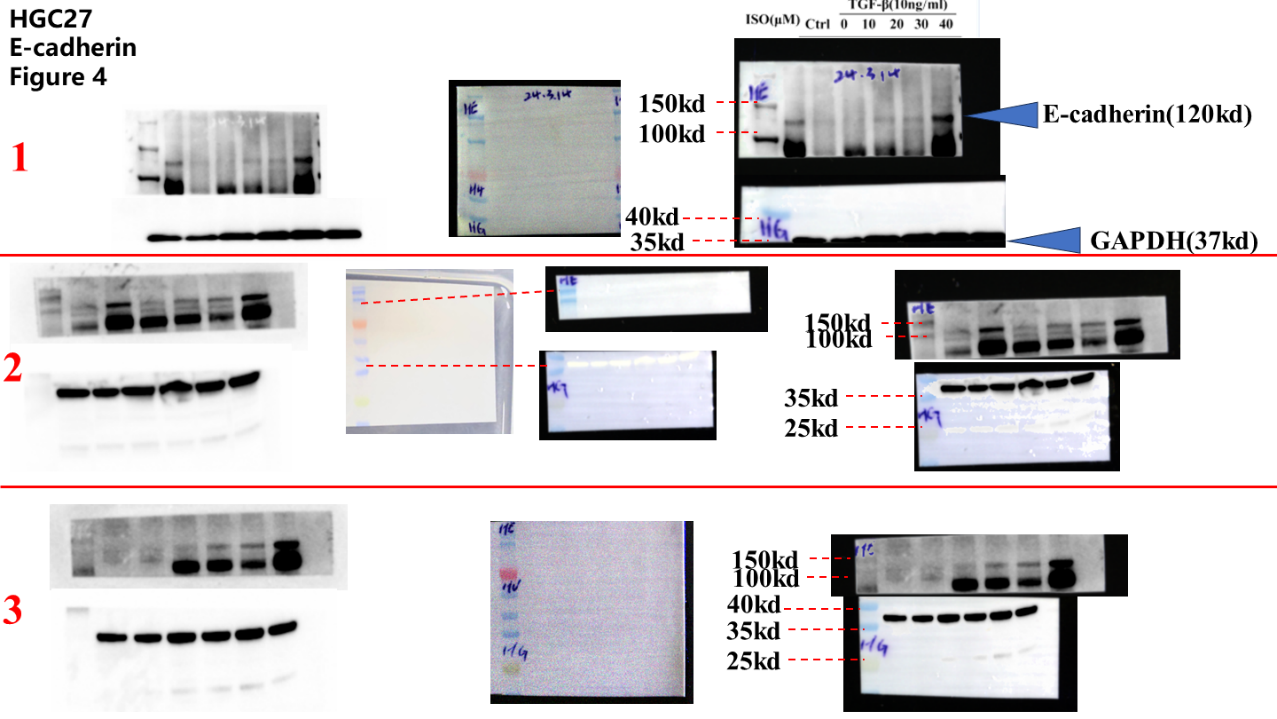


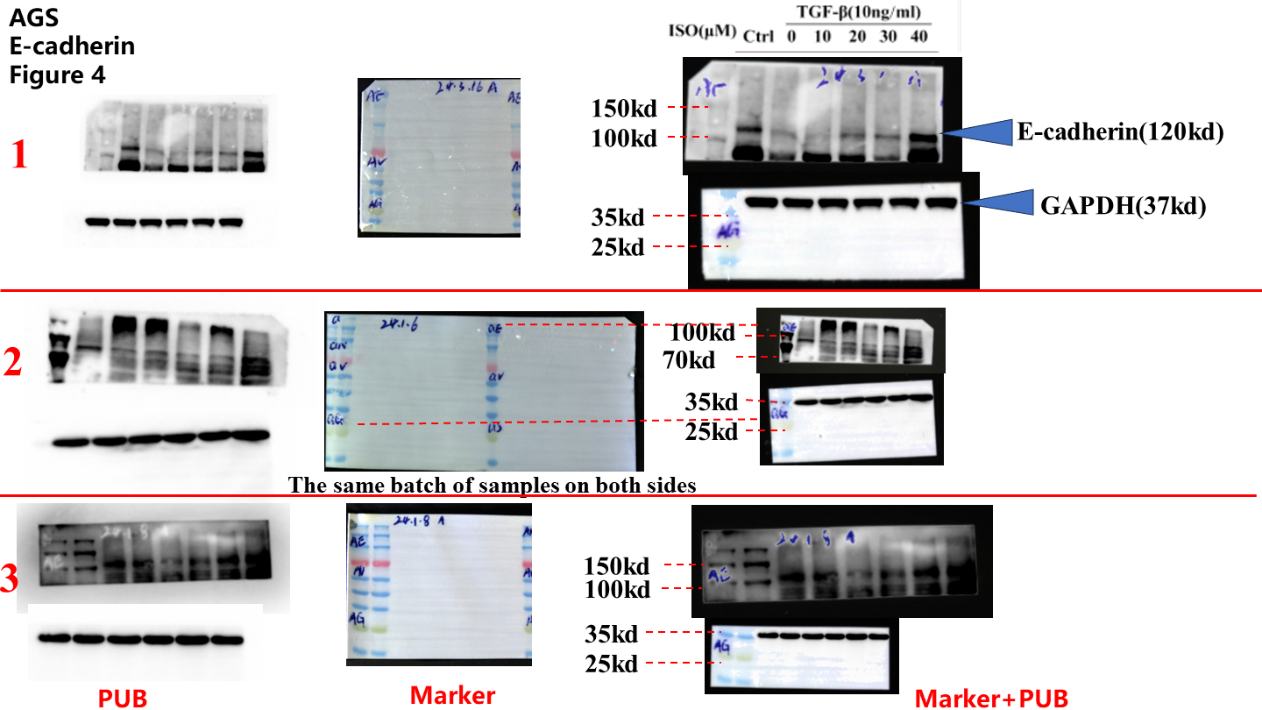


# Original western blots for Figure 4(N-Cadherin)


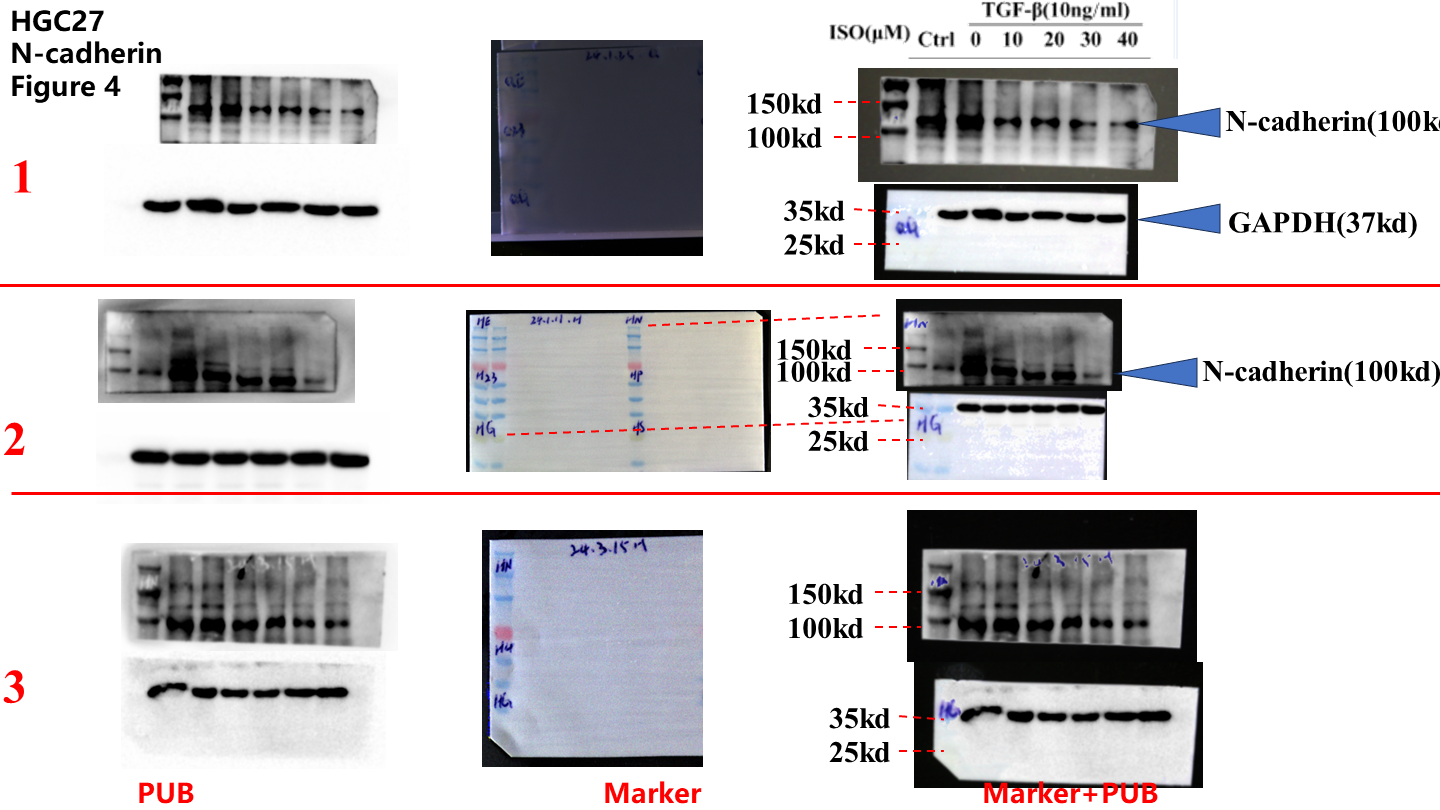

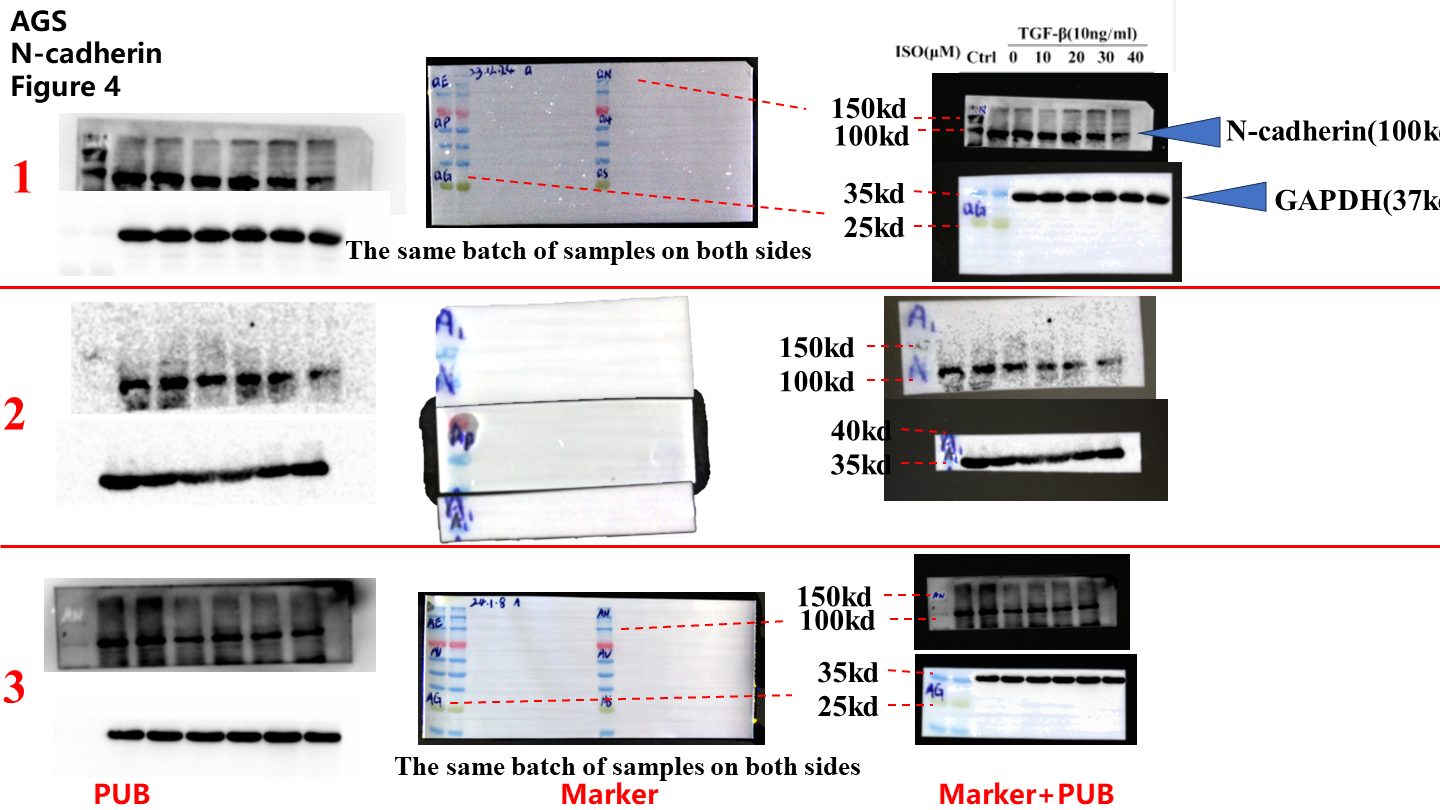


# Original western blots for Figure 4(Vimentin)


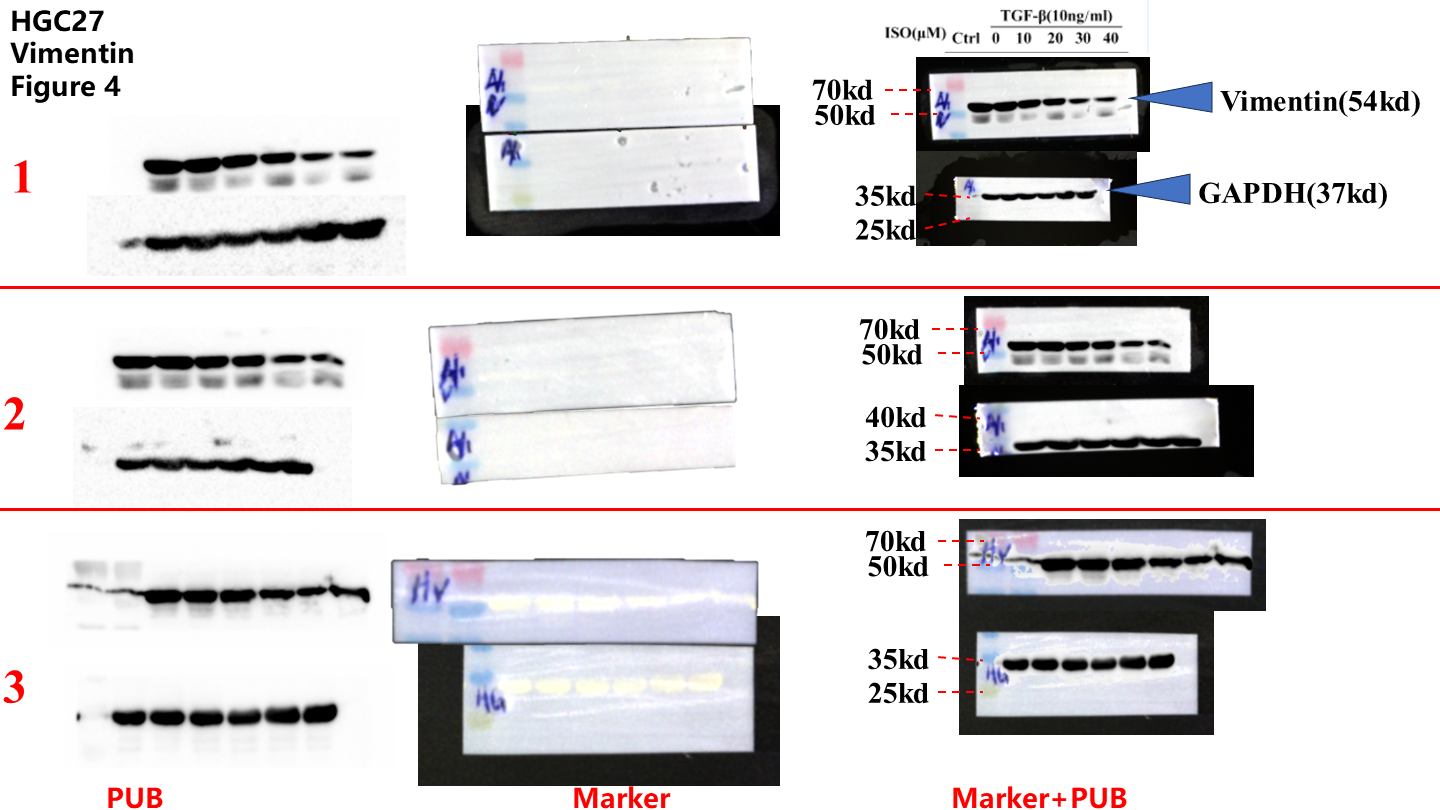


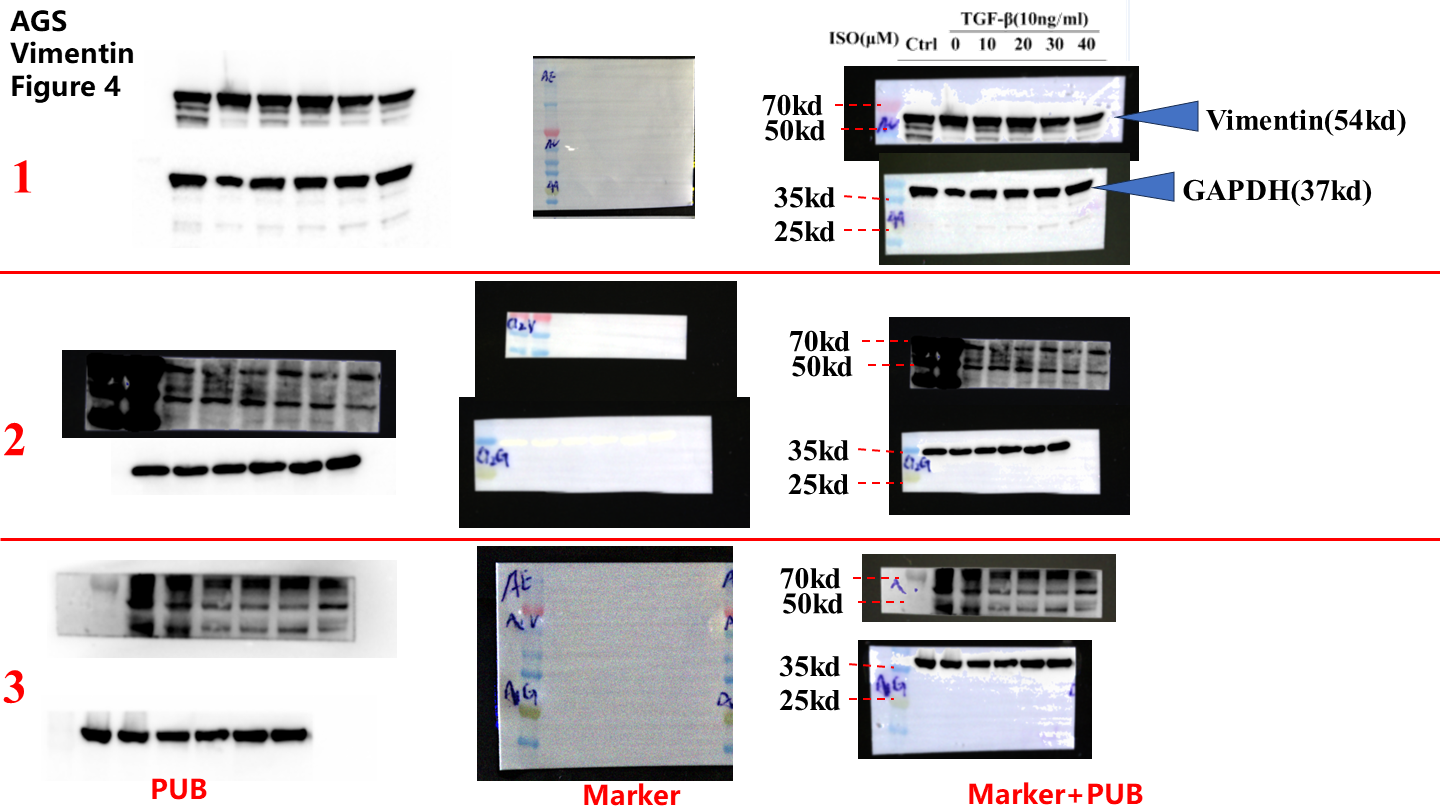


# Original western blots for Figure 4（SNAI1）


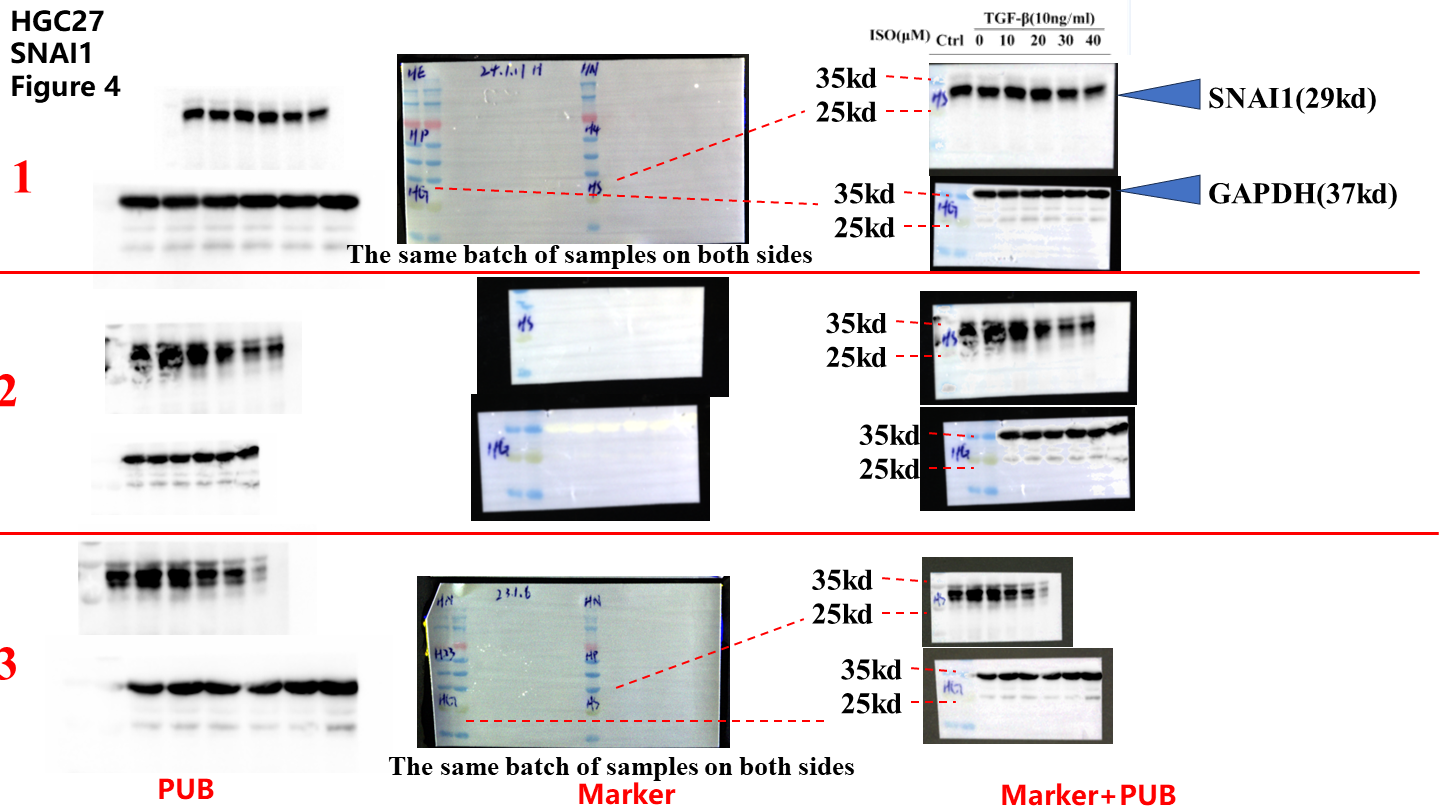


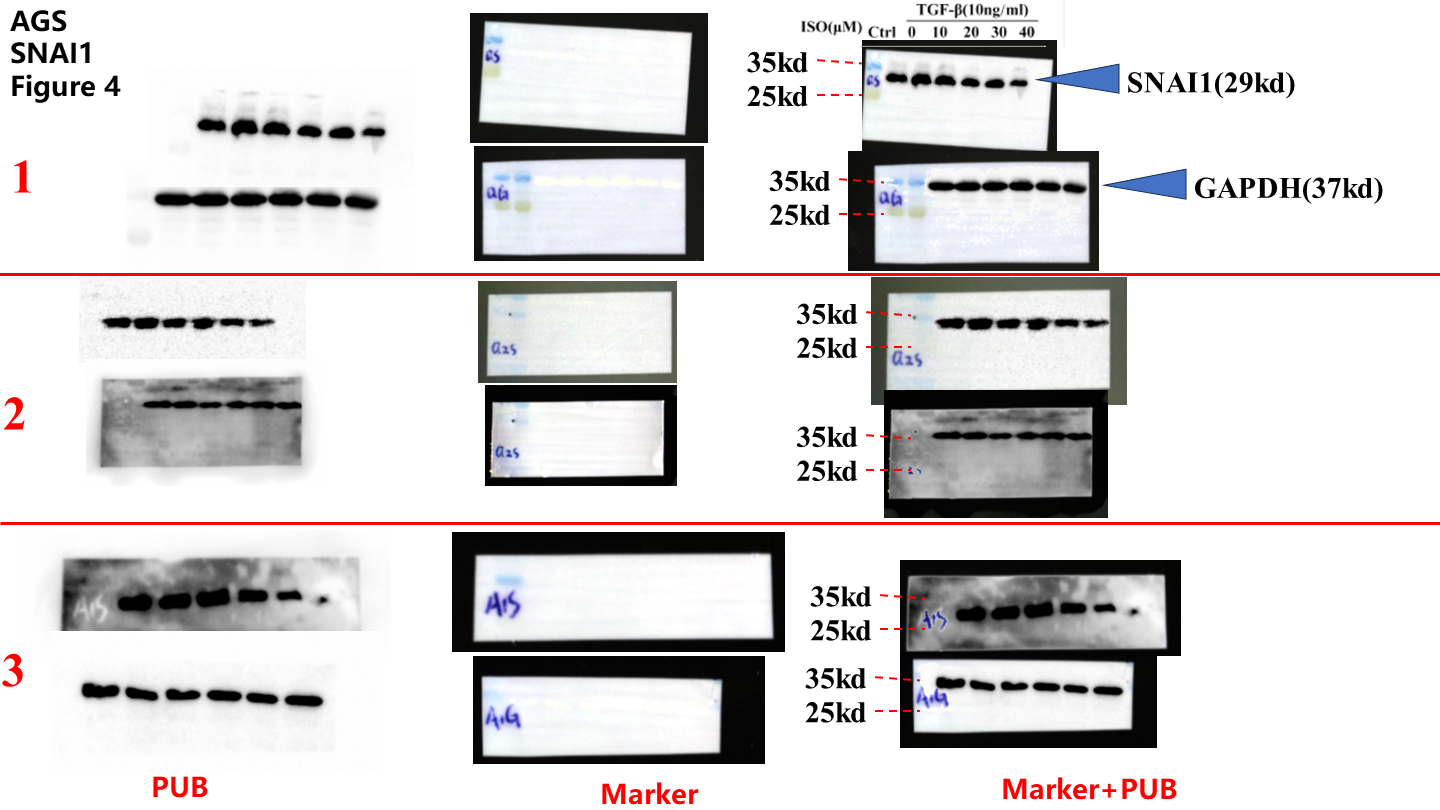


# Original western blots for Figure 4（P-Smad2; TGF-β stimulation 1h）


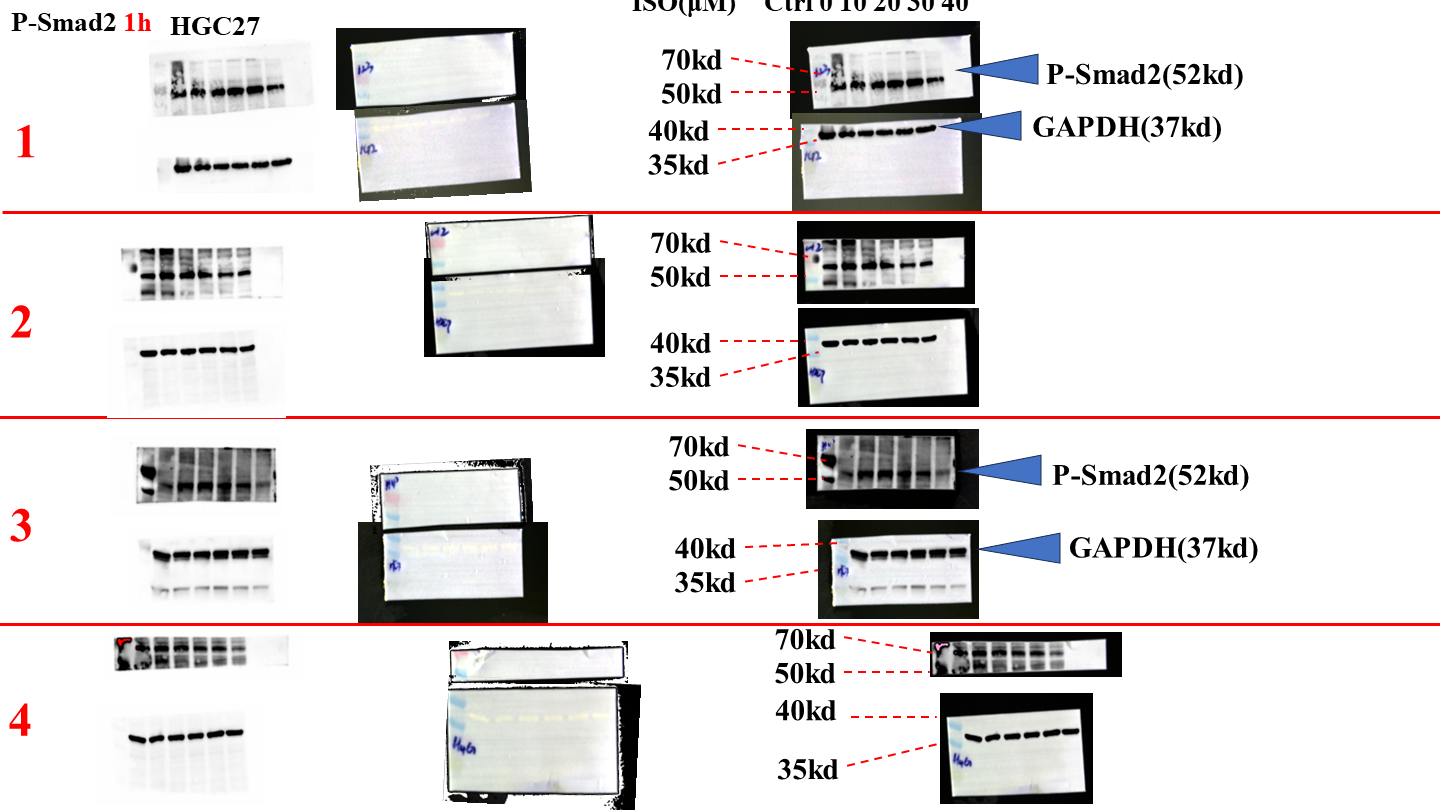


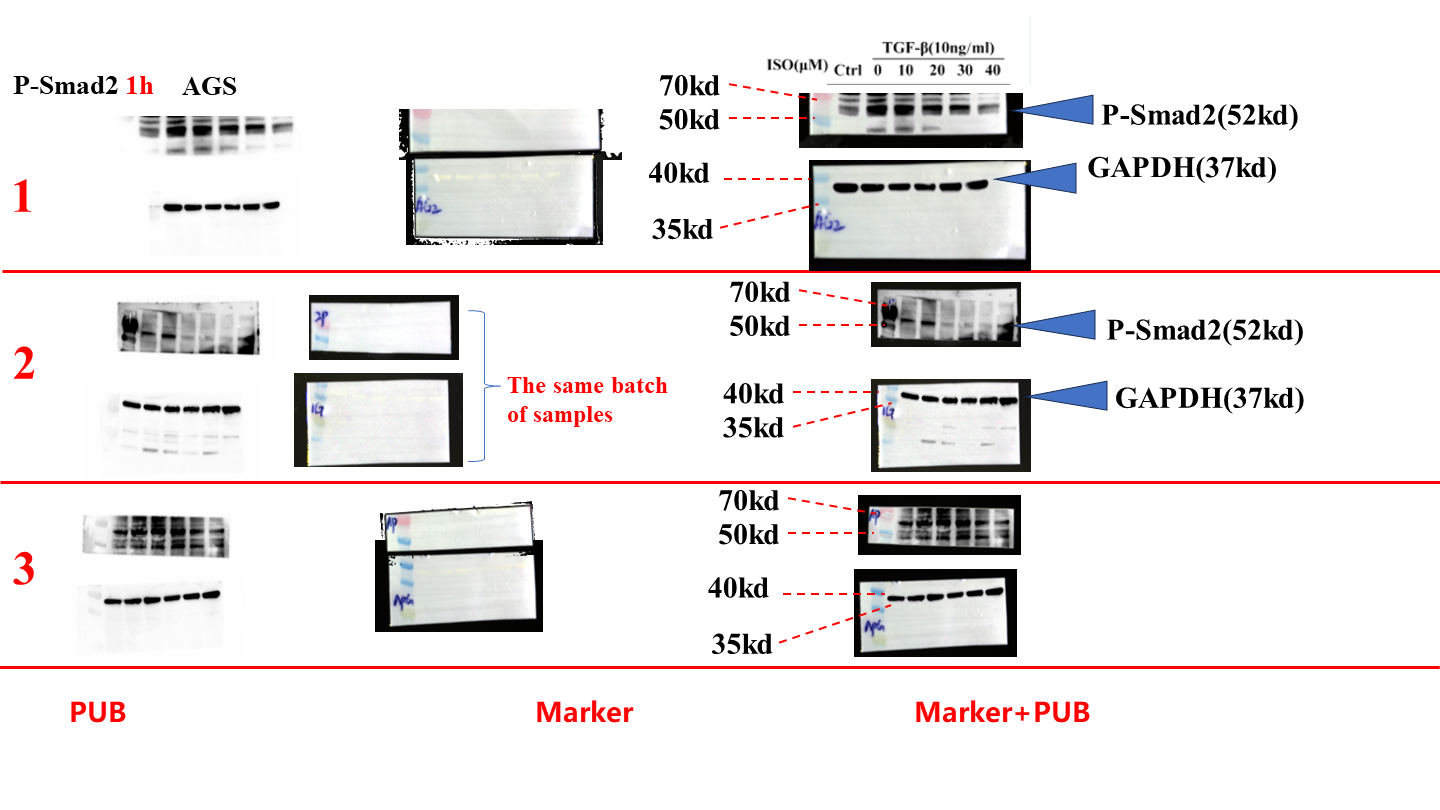


# Original western blots for Figure 4（Smad2;TGF-β stimulation 1h）


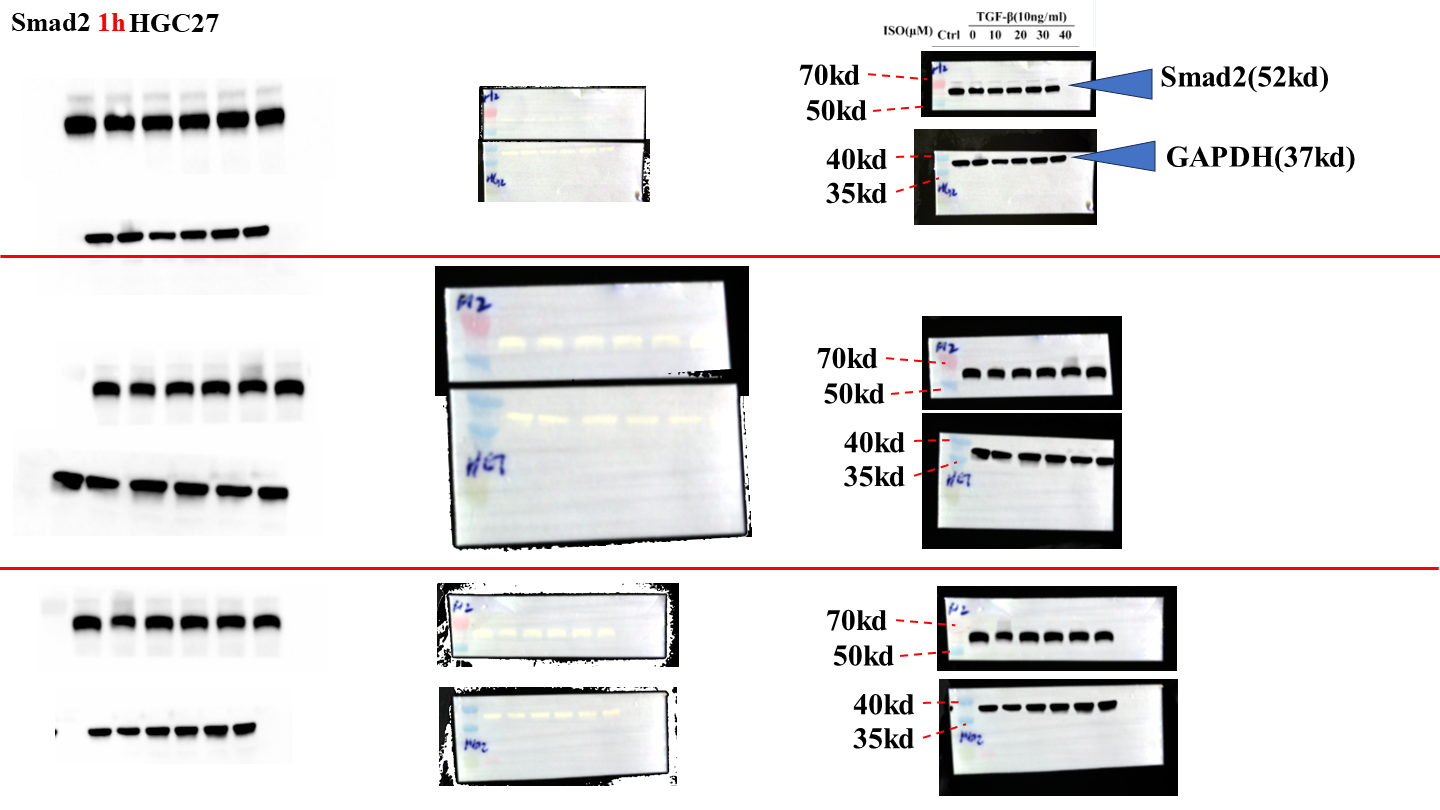


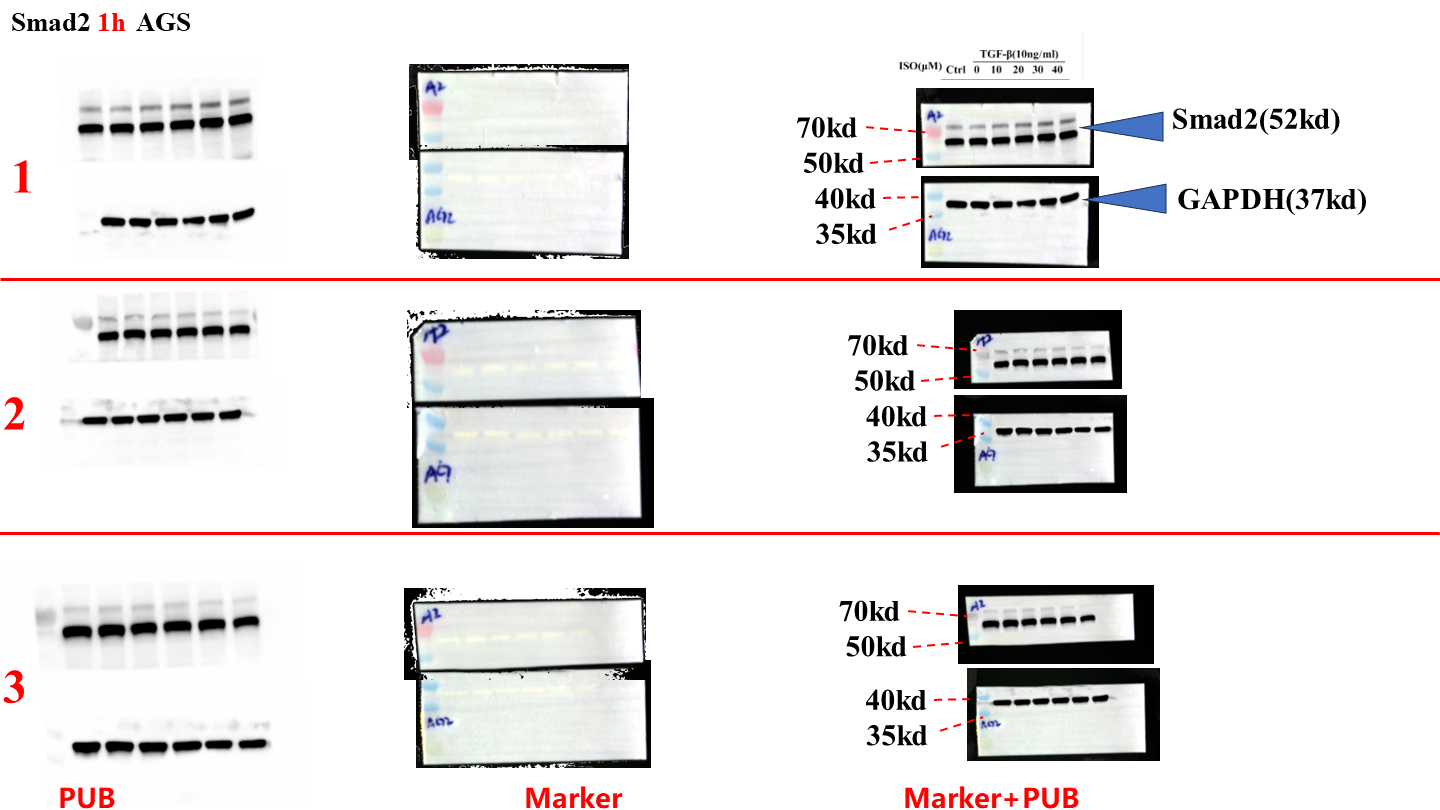


# Original western blots for Figure 4（Smad4; TGF-β stimulation 1h）


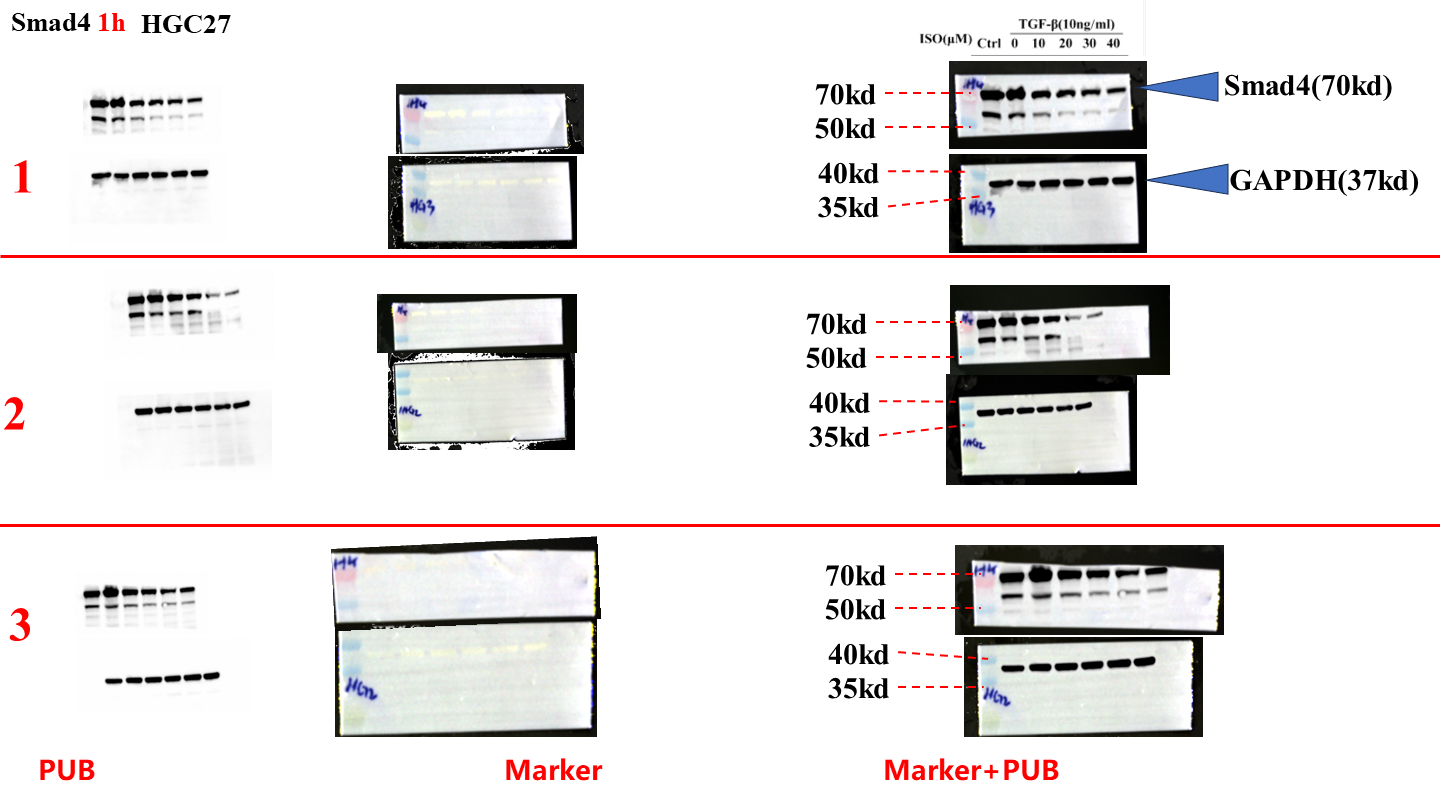


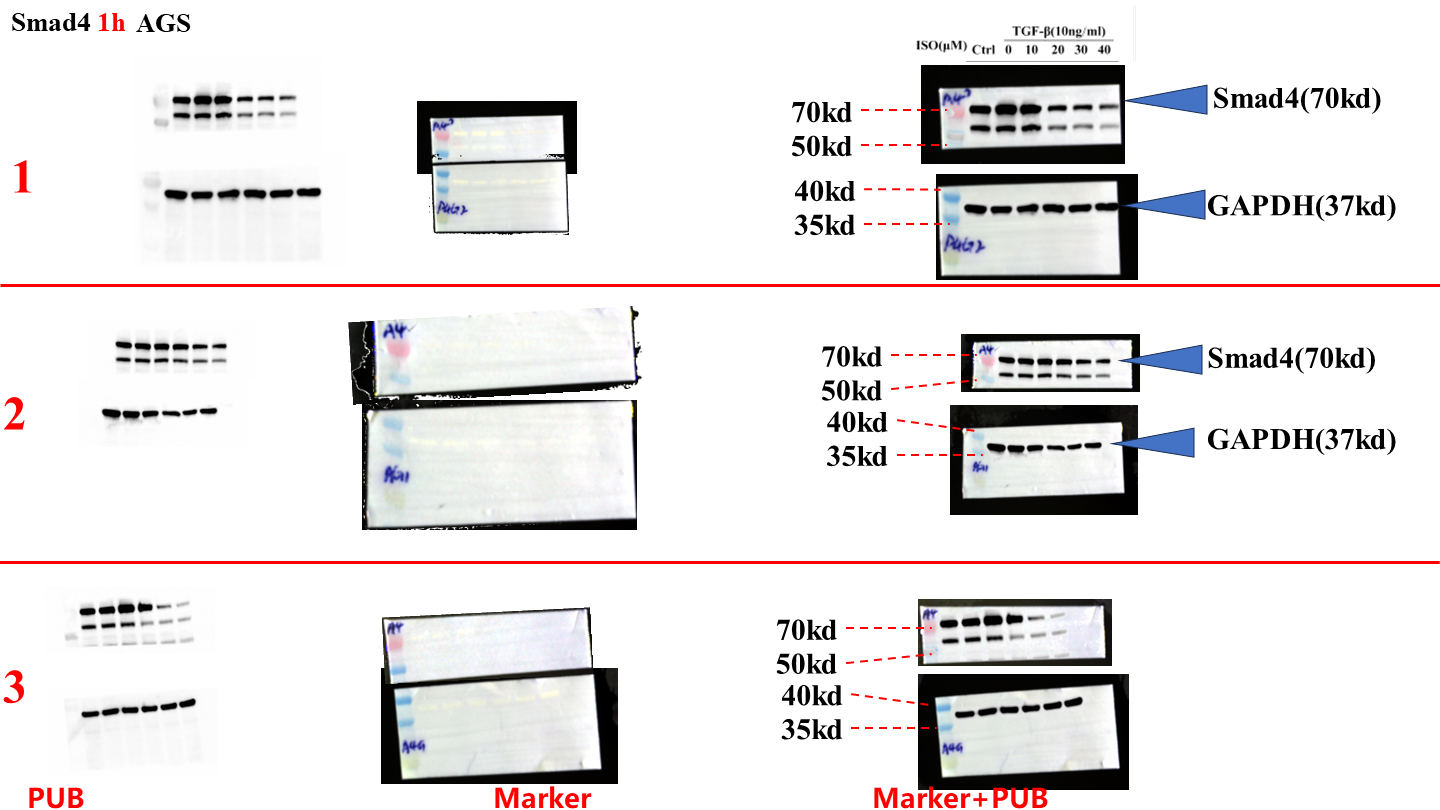


# Original western blots for Figure 4（P-Smad2; TGF-β stimulation 24h）


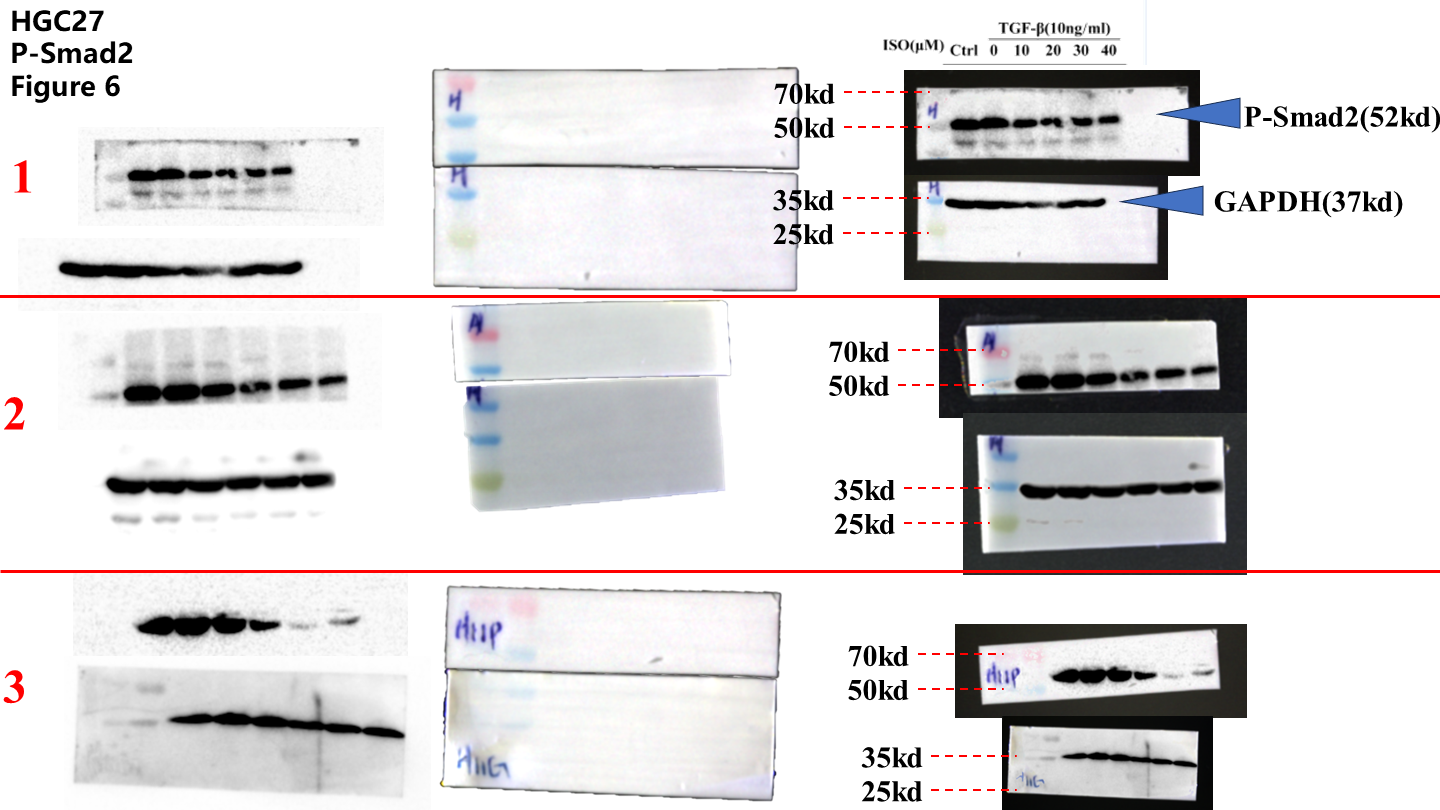


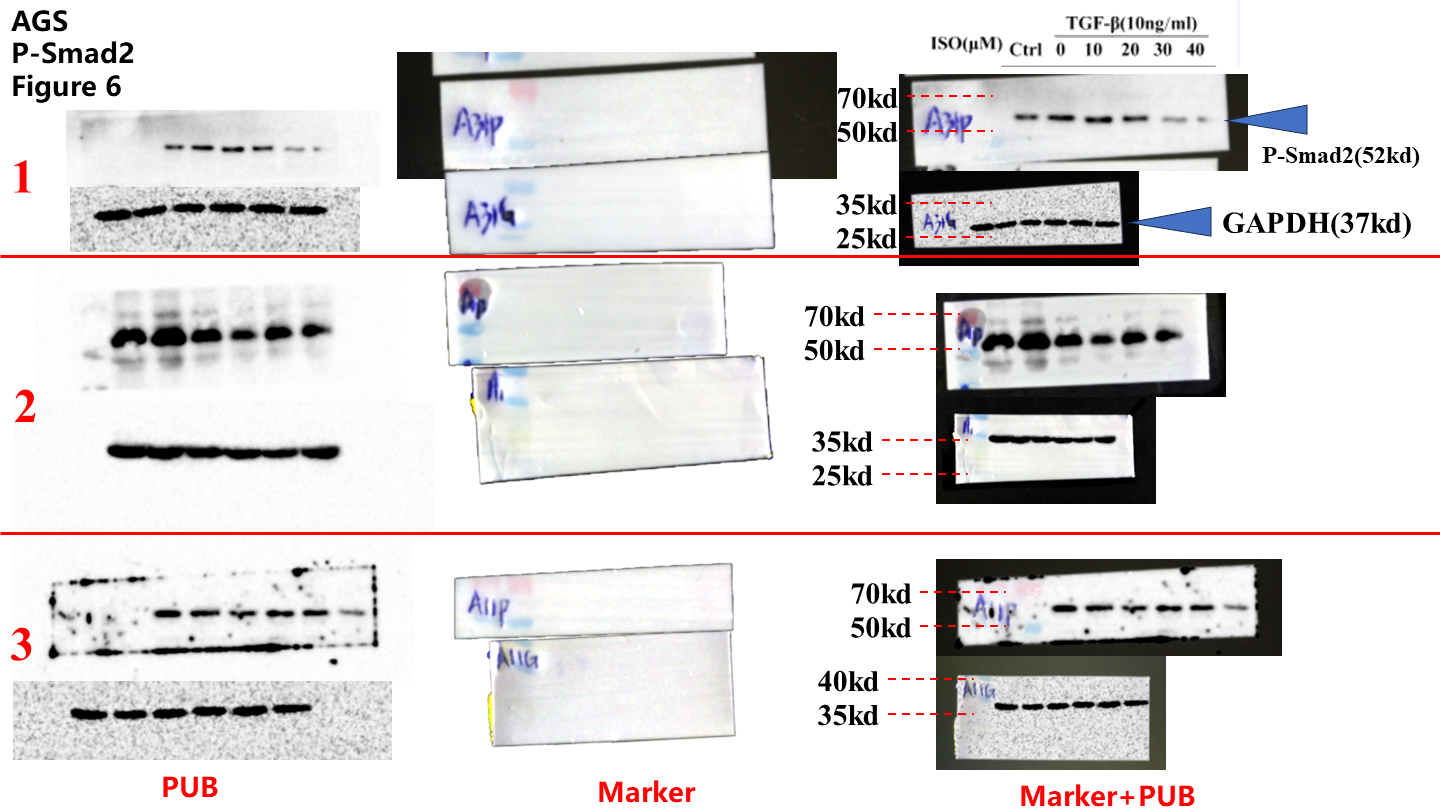


# Original western blots for Figure 4（Smad2; TGF-β stimulation 24h）


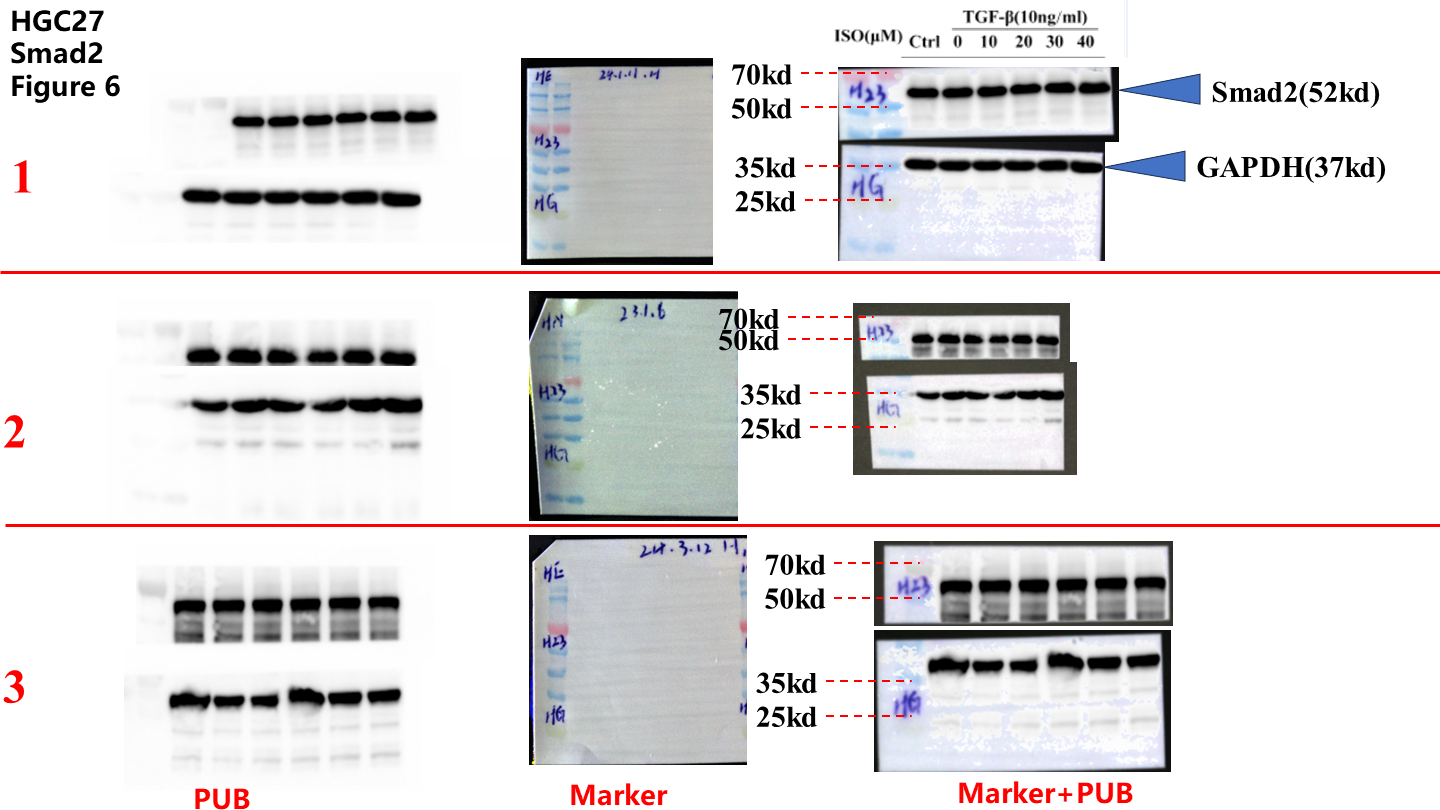


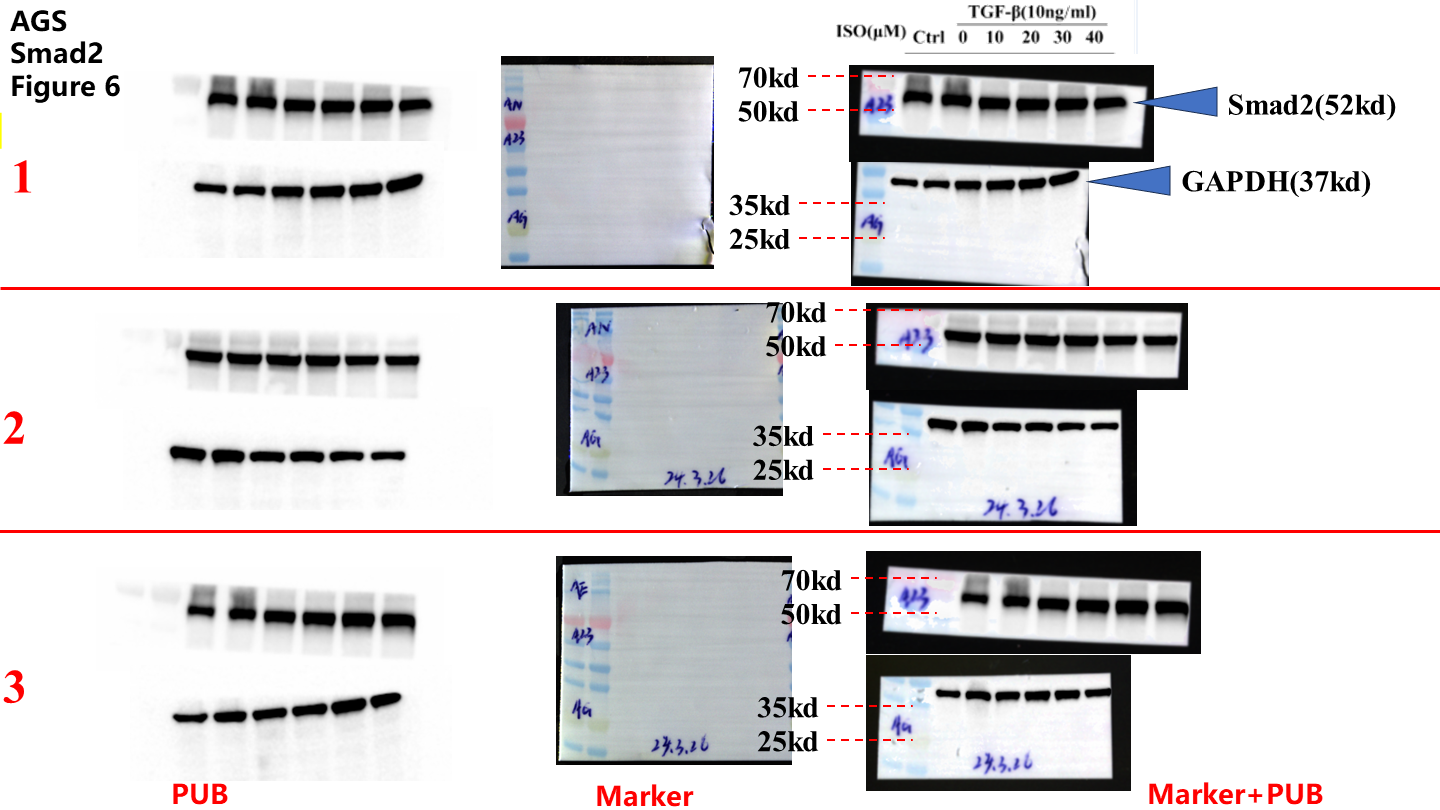


# Original western blots for Figure 4（Smad4; TGF-β stimulation 24h）
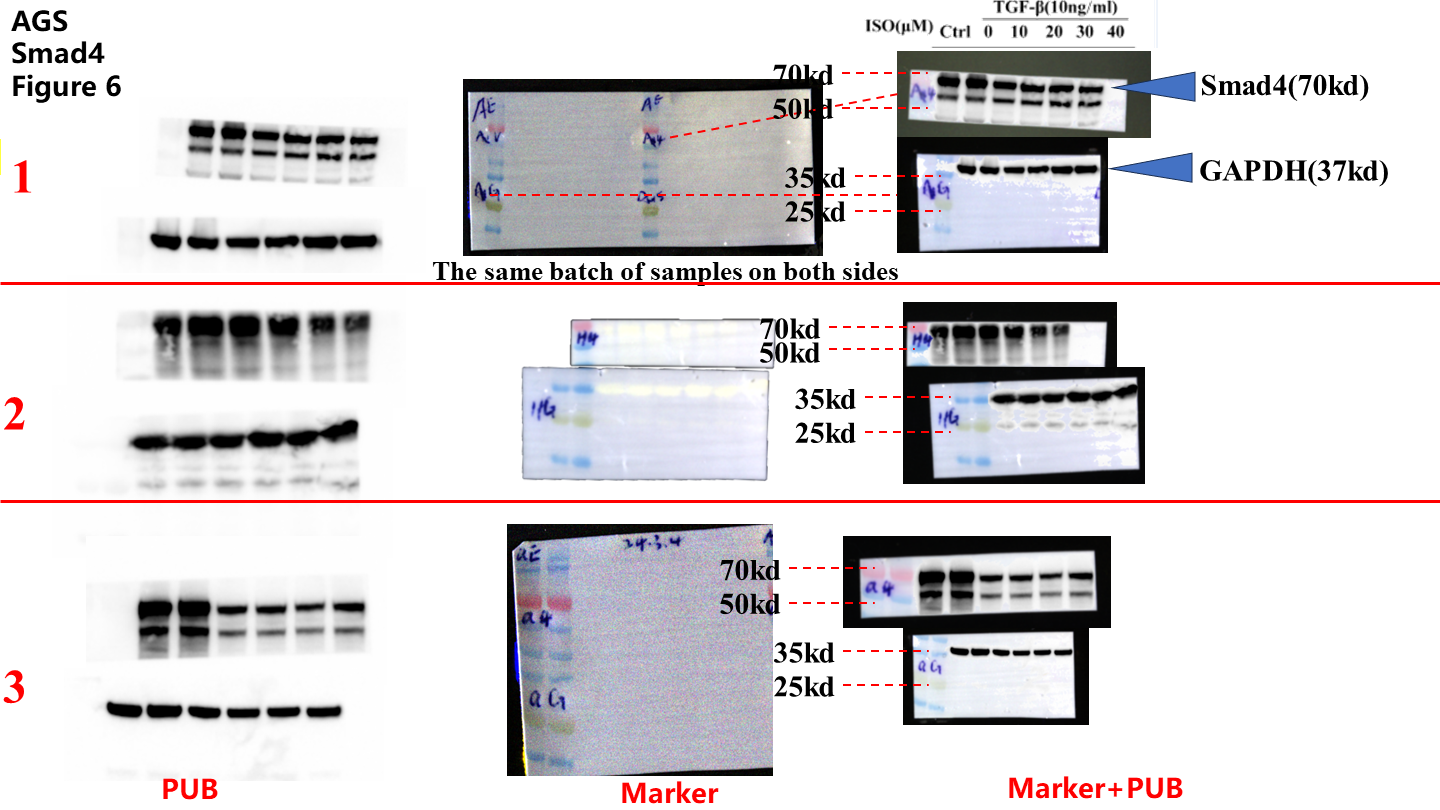


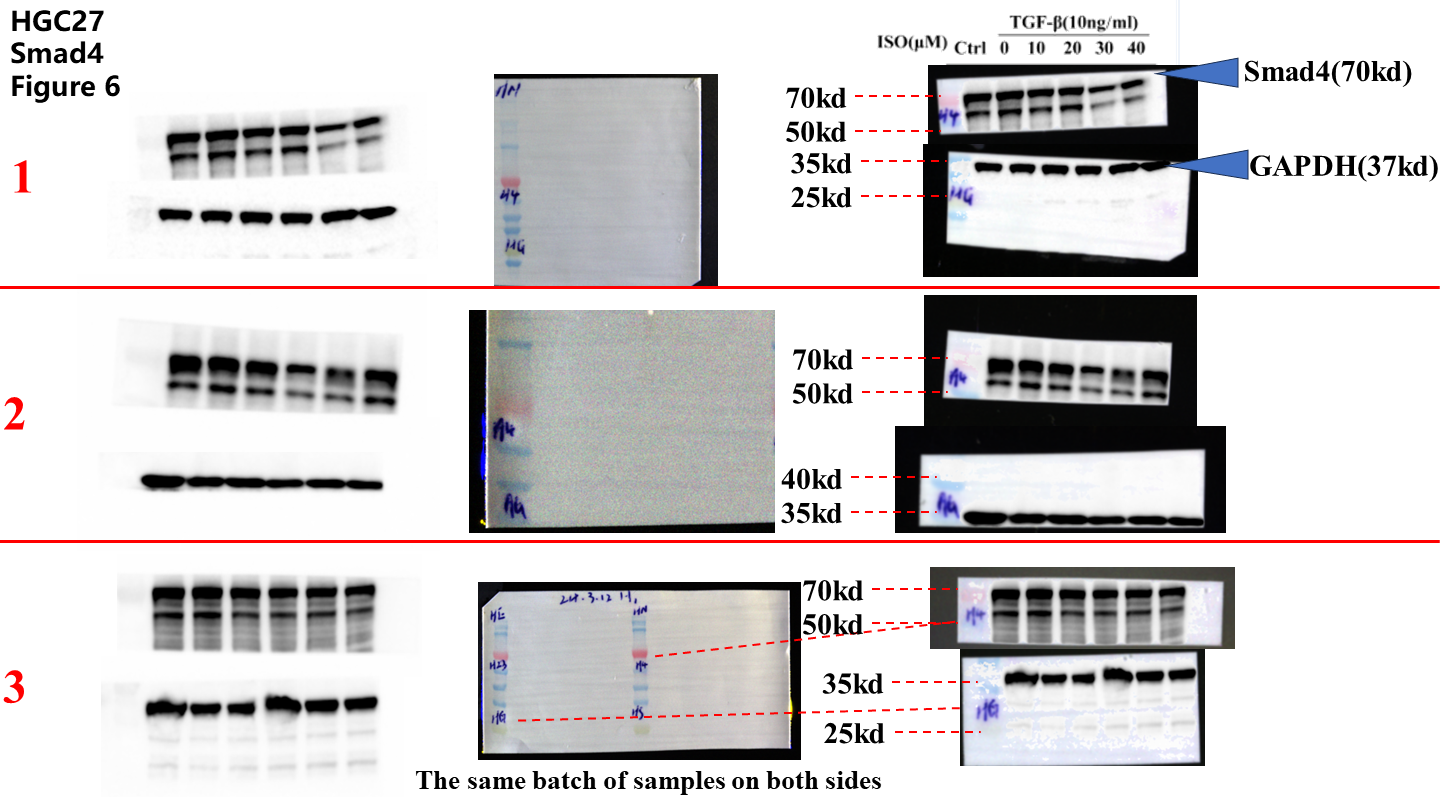


# Original western blots for Figure 4（siTGFBR1）


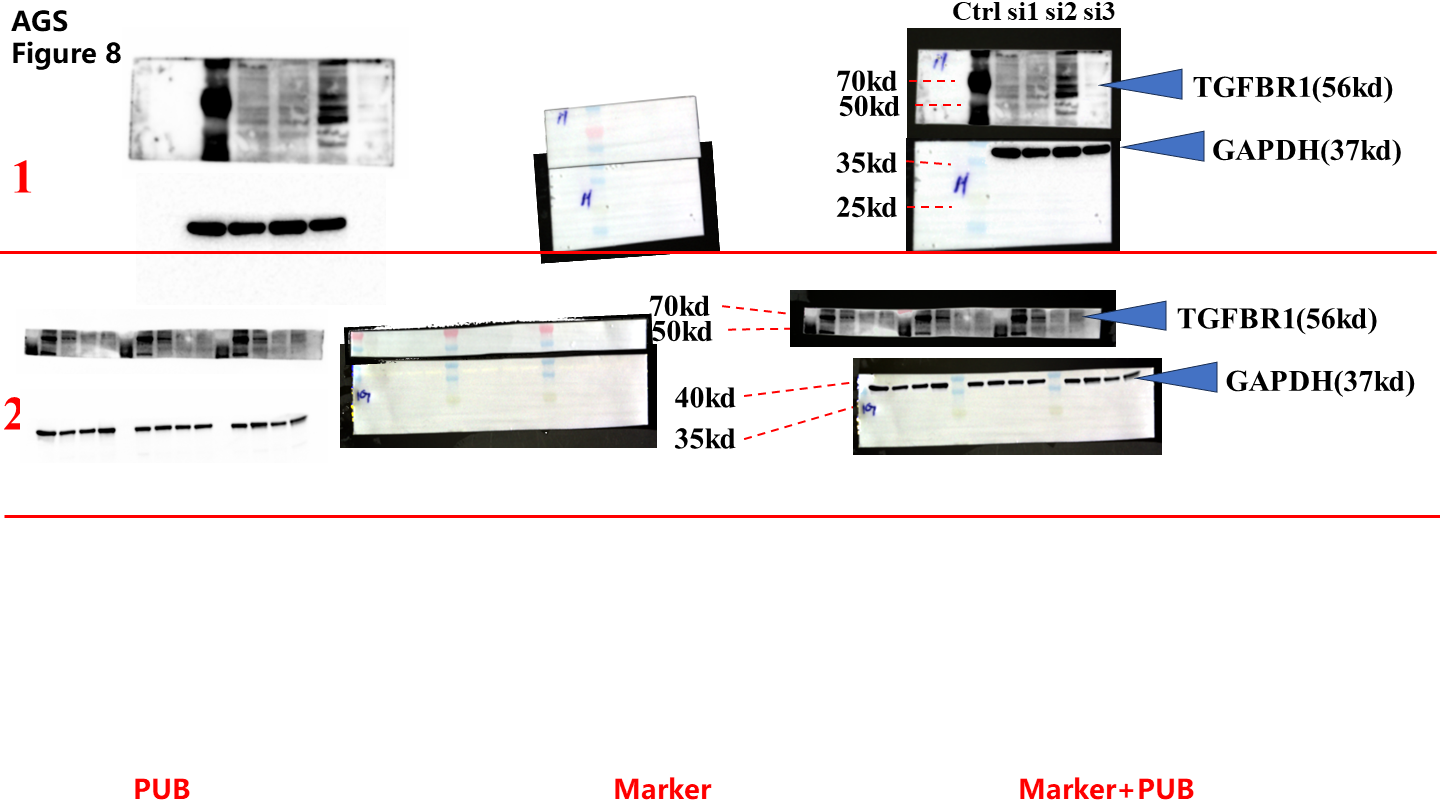

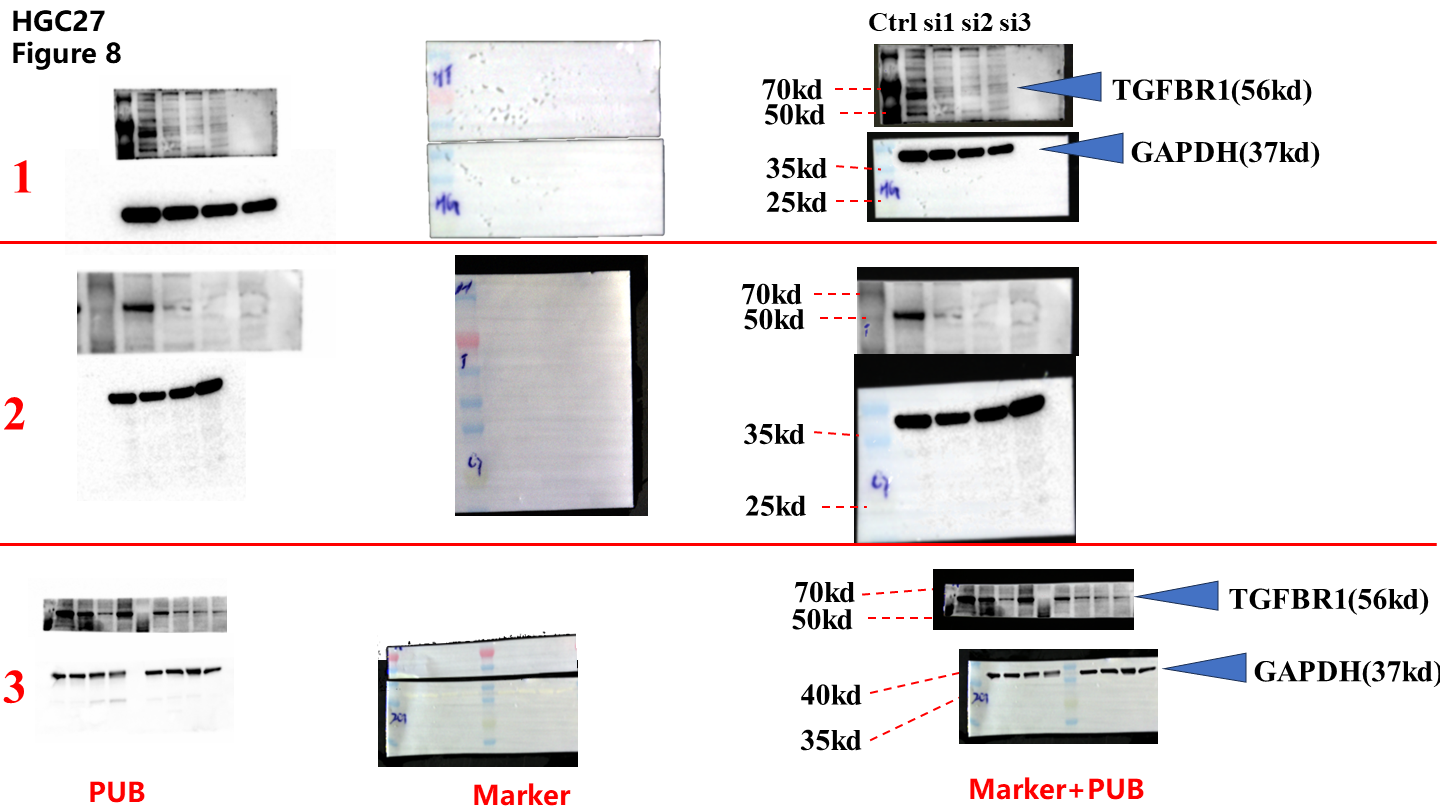


# Original western blots for Figure


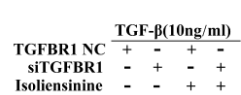

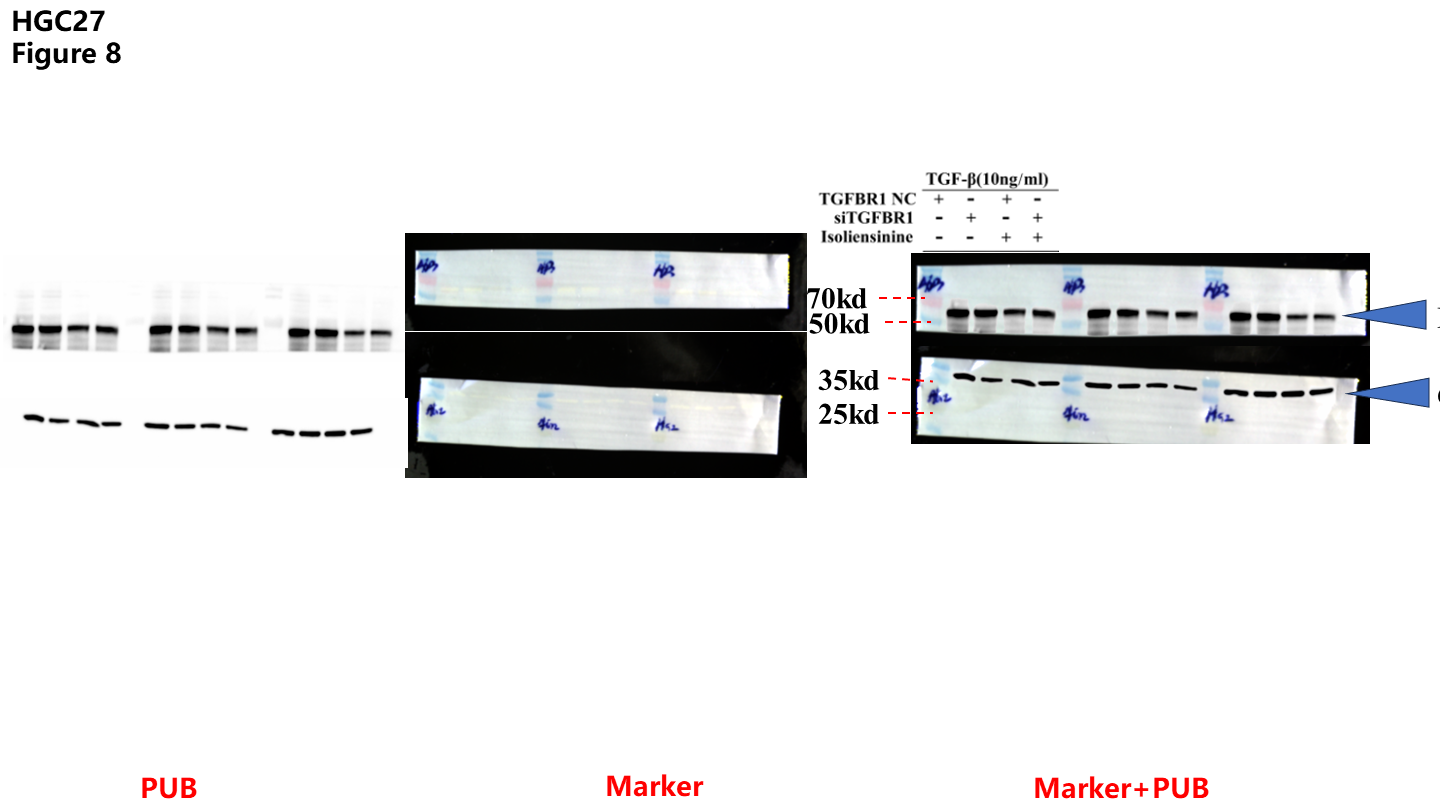


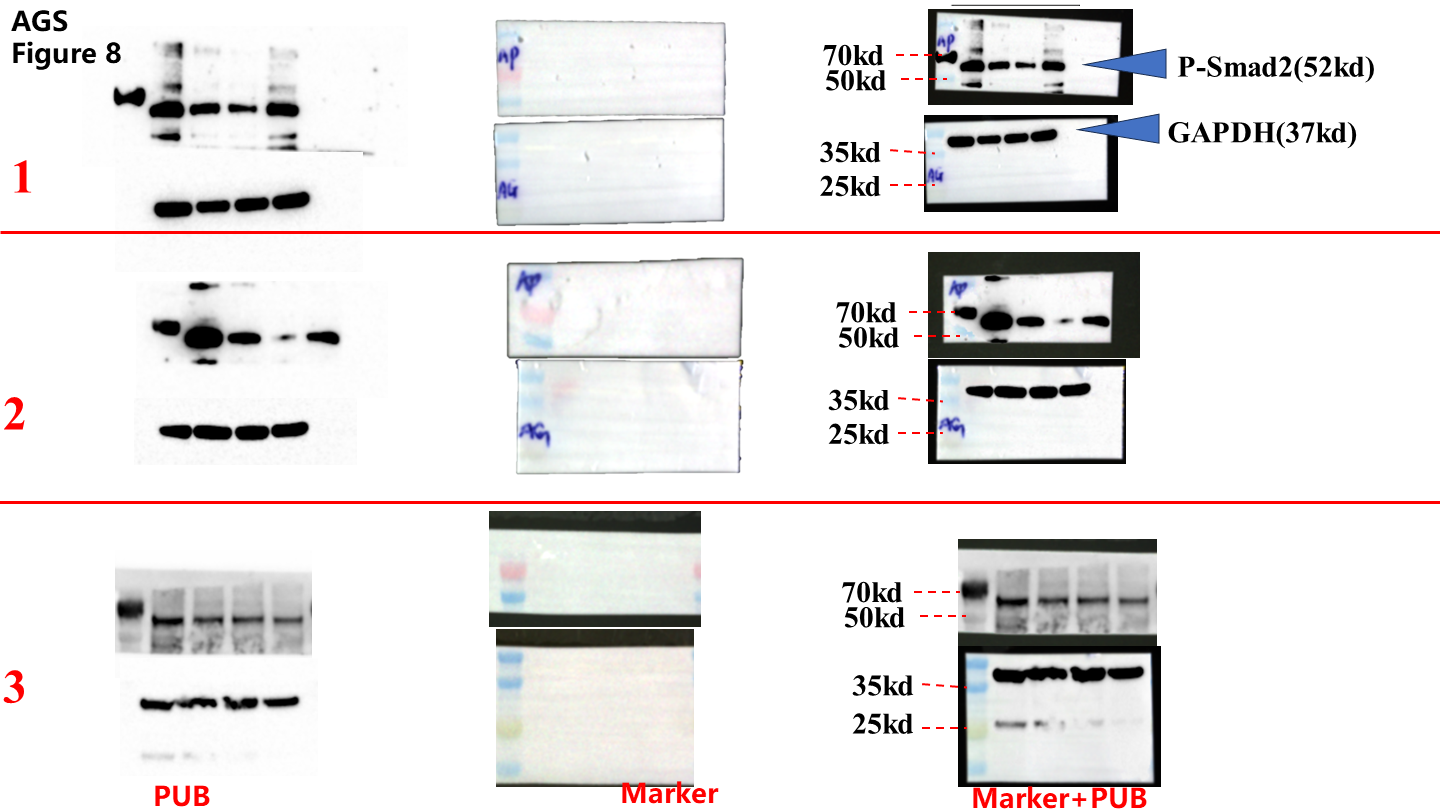


# Original western blots for Figure 4
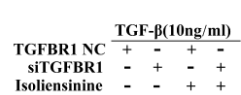


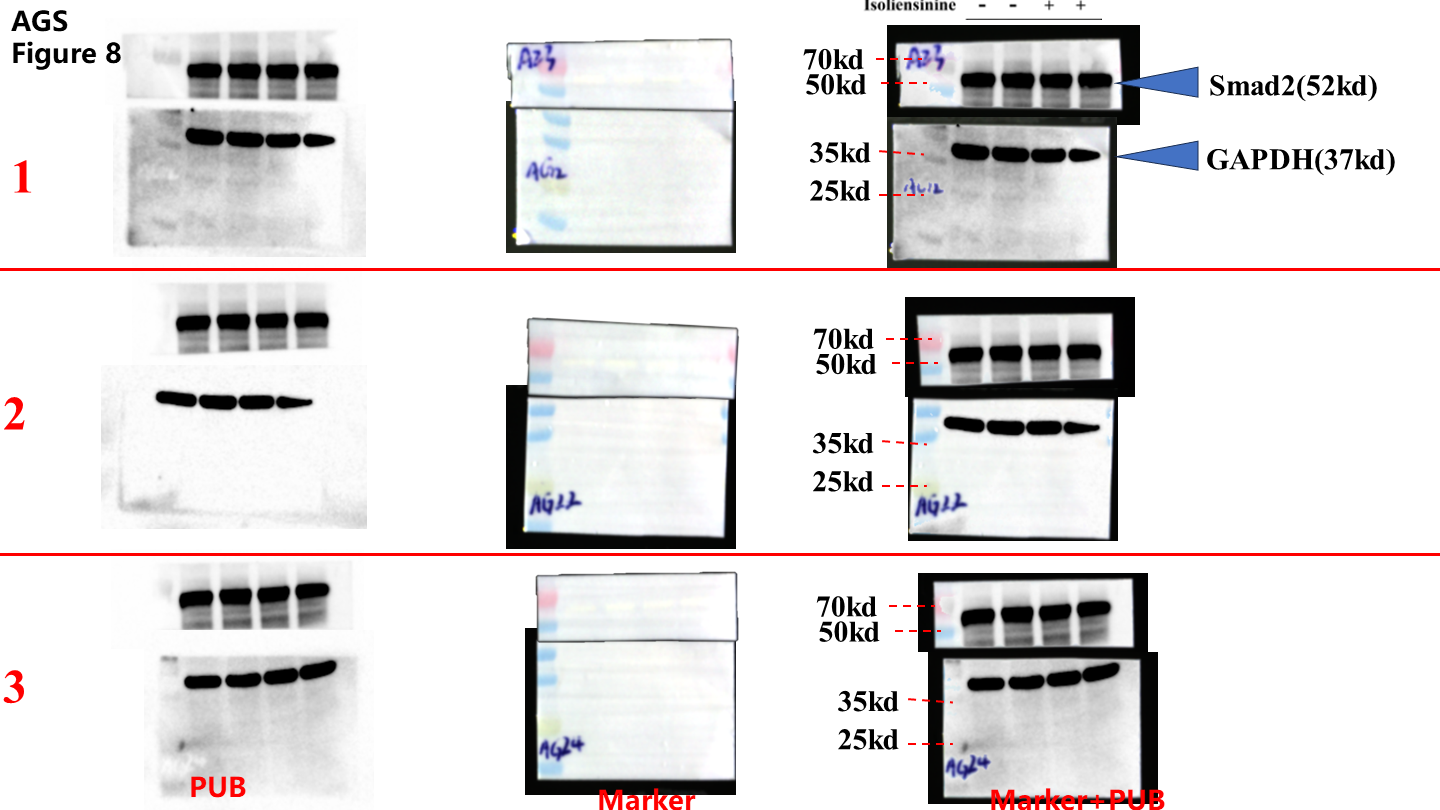


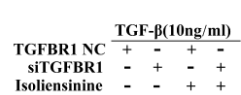


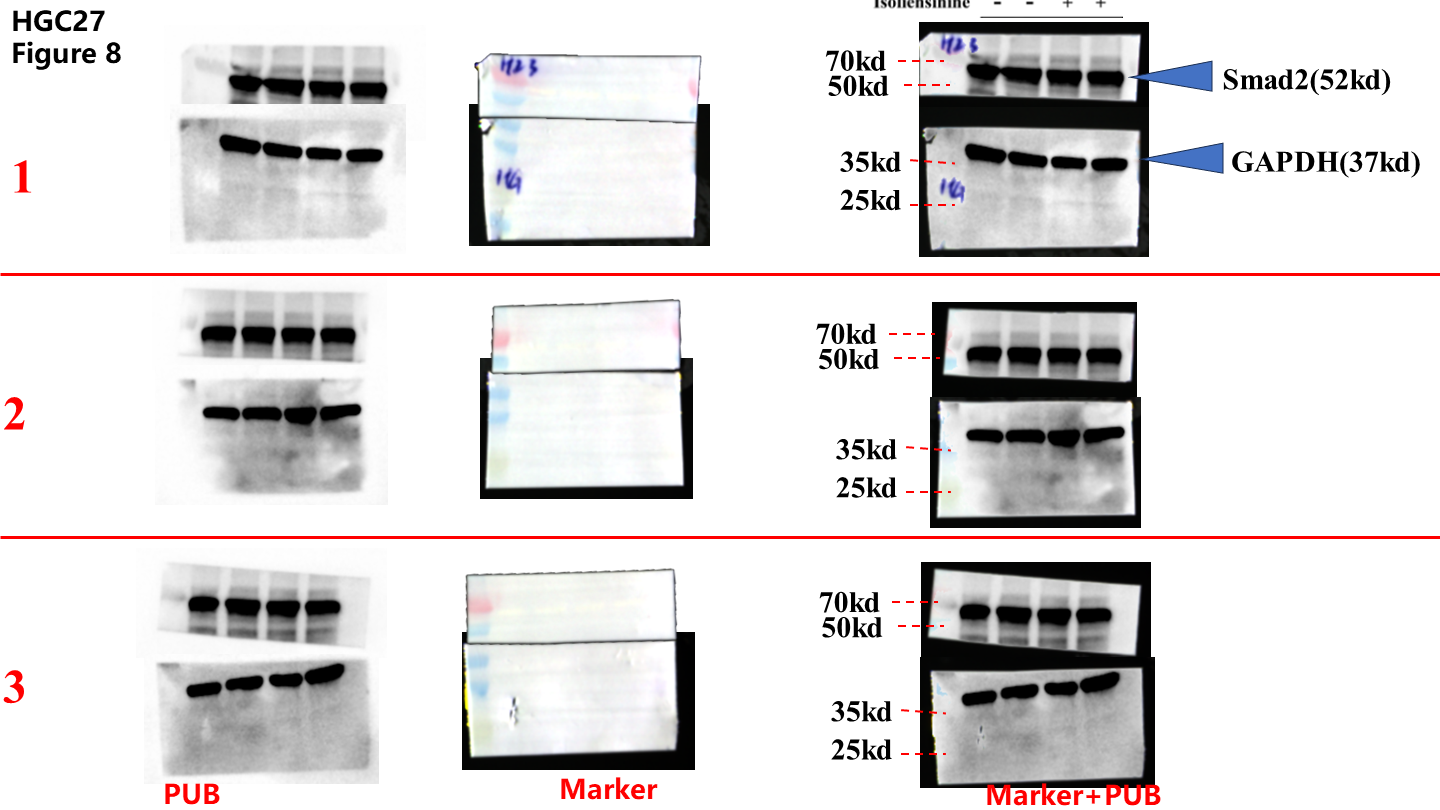


# Supplementary Figures


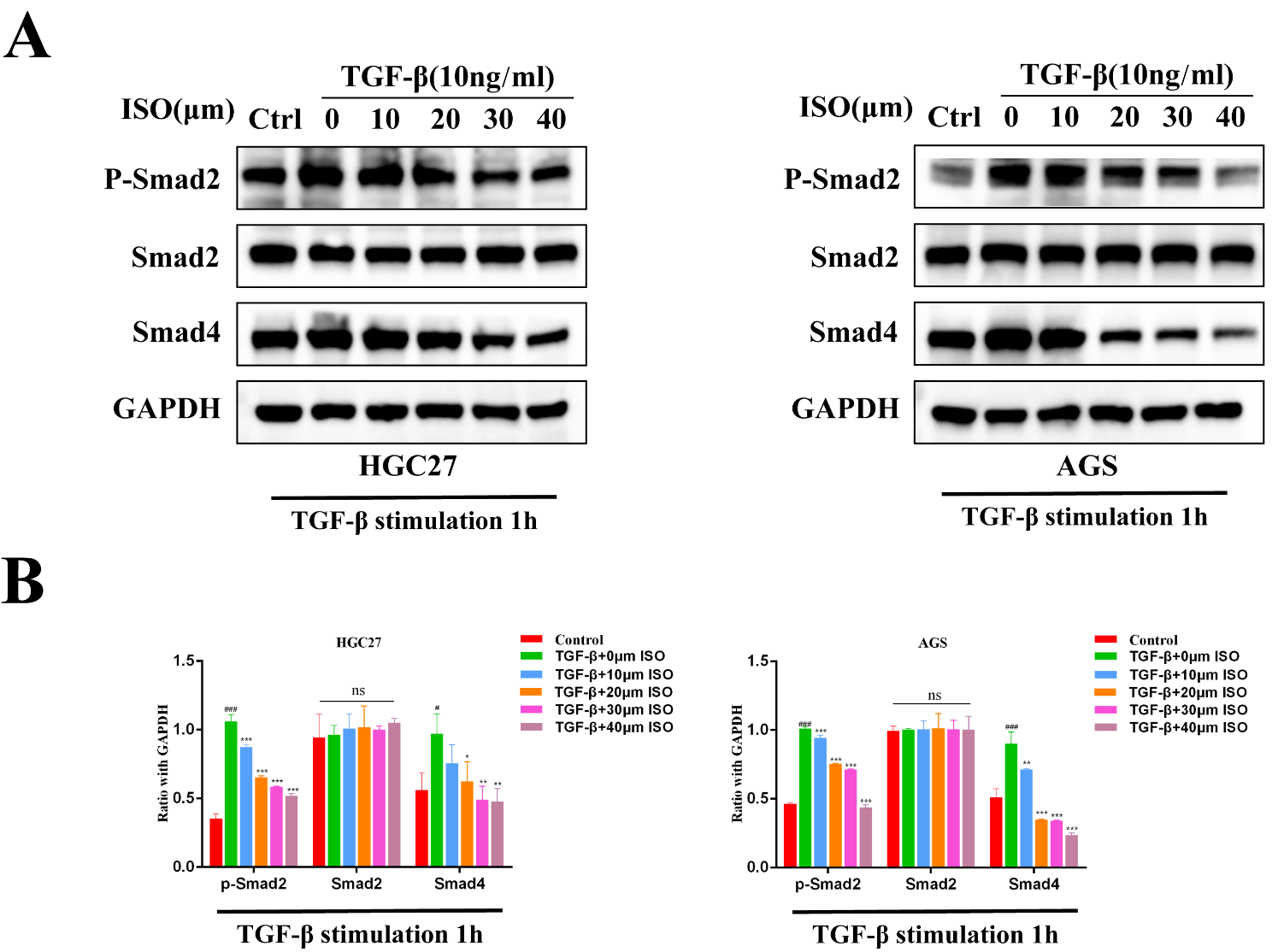


**Supplementary Figure 1.**

Western blotting assesses the impact of ISO on the proteins involved in the TGF-β-Smad pathway. (A)Pretreatment with different concentrations of ISO (0, 10, 20, 30, 40 μM) for 2 h, followed by TGF-β induction for 1 h, and Western blotting to detect the levels of p-Smad2, Smad2, and Smad4in HGC27 cells and AGS cells. (B) The HGC27 and statistical data pertain to the relative protein expression in AGS cells. Results are expressed as means ± SEM, n = 3. ** *P* < 0.01, *** *P* < 0.001and ns, not significant (P > 0.05) when compared to the DMSO group. # *P* < 0.05 and ### *P* < 0.001 when compared to Control group.


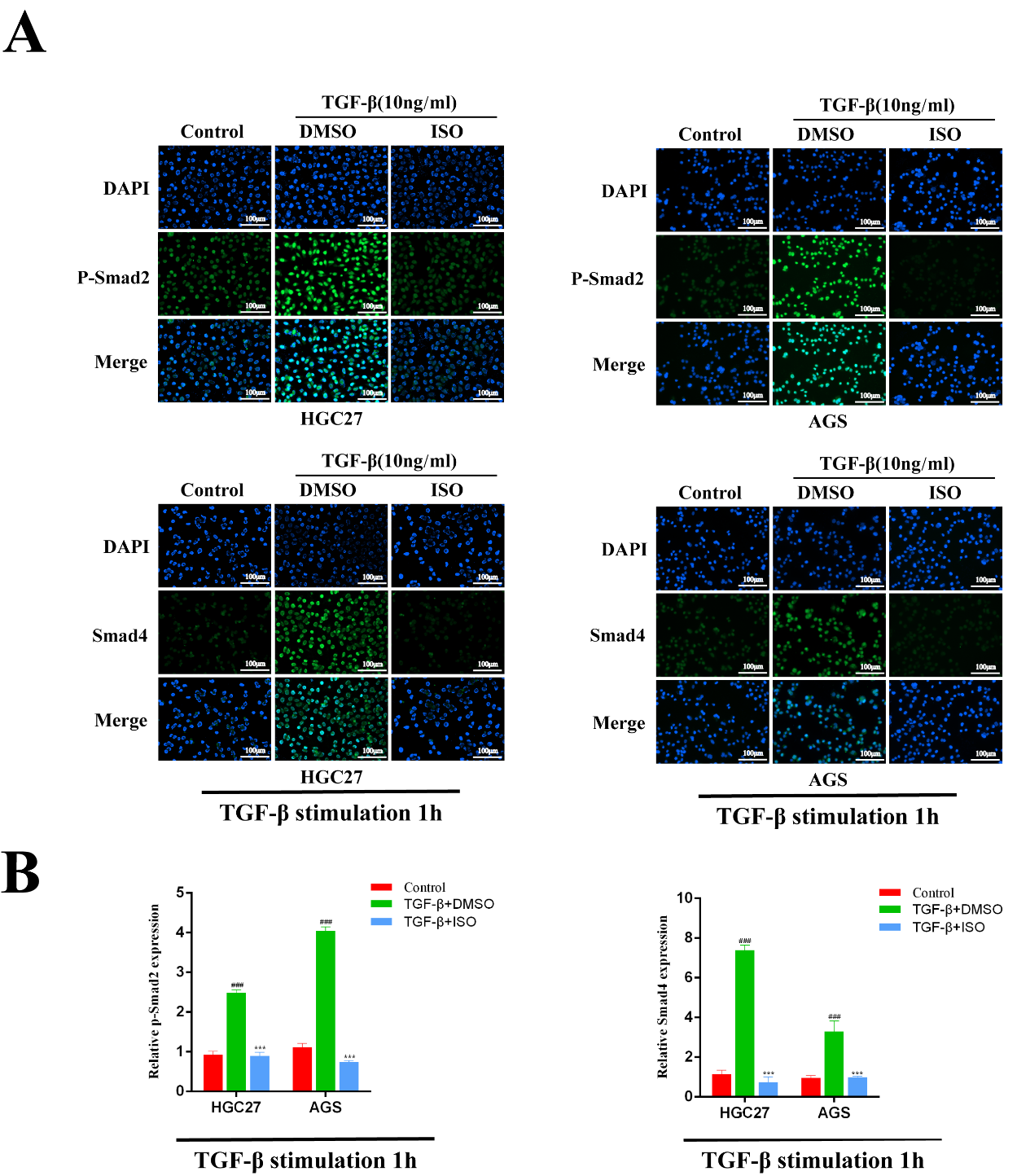


**Supplementary Figure 2.**

Immunofluorescence was used to evaluate the influence of ISO on the proteins relevant to the TGF-β-Smad pathway. (A) Immunofluorescence detection of p-Smad2, Smad2, and Smad4 levels in HGC27 and AGS cells after two h pretreatment with 10 μM ISO for two h and TGF-β induction for 1h. (B) Immunofluorescence was used to detect the impact of ISO on the proteins involved in the TGF-β-smad pathway. Results are expressed as means ± SEM, n = 3. *** *P* < 0.001, compared to the DMSO group. ### *P* < 0.001 when compared to Control group.

**A**


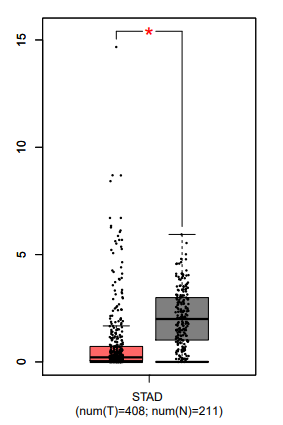
ALB
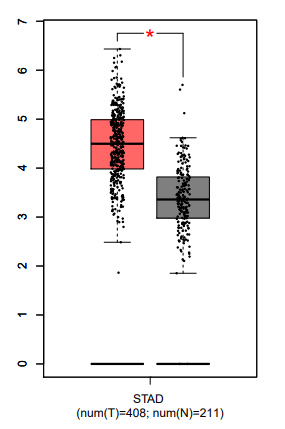
CASP3
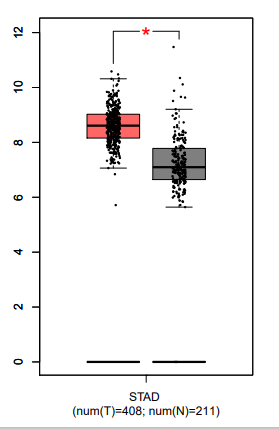
 HSP90AA1
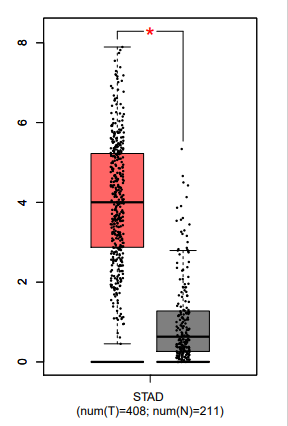
 MMP9
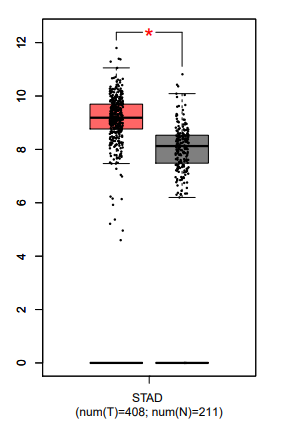
GSTP1
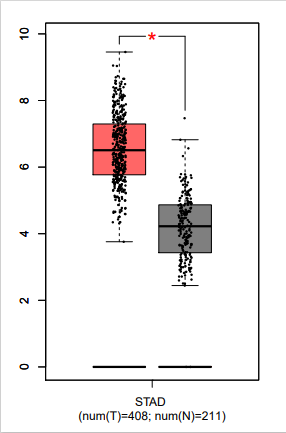
STAT1


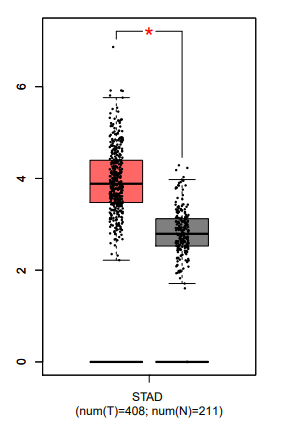
XIAP
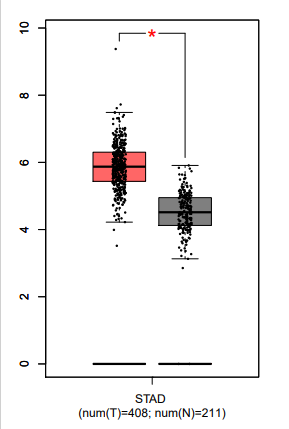
PARP1
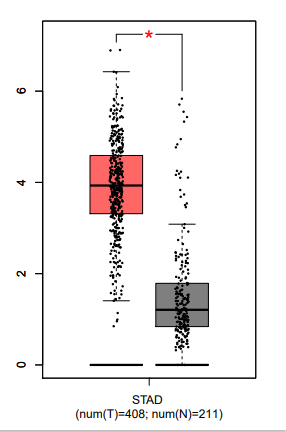
 VDR
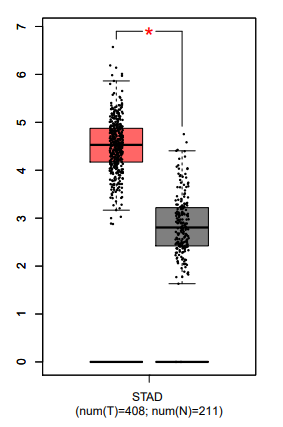
 GSK3B
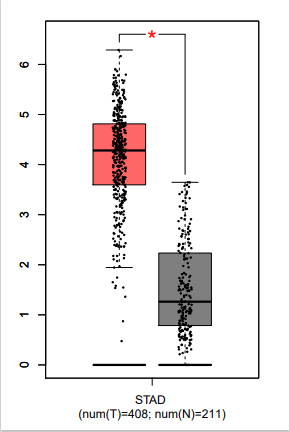
CCNA2
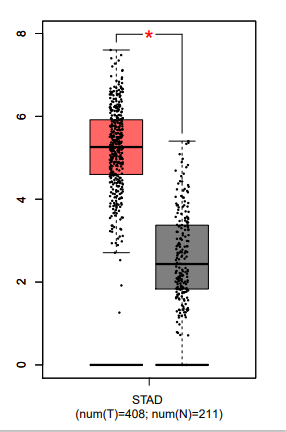
TYMS
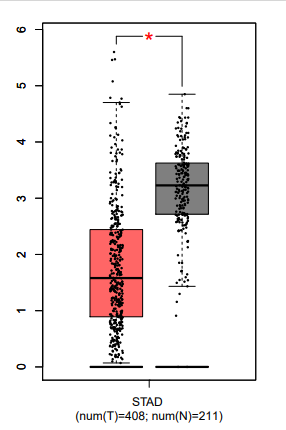
KIT
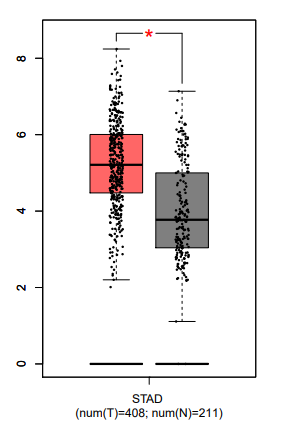
PDGFRB
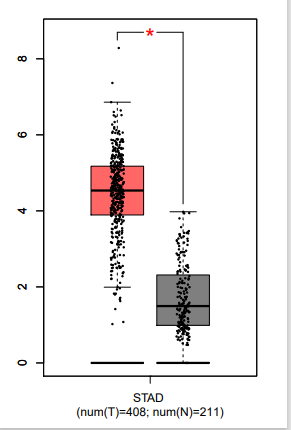
 AURKA
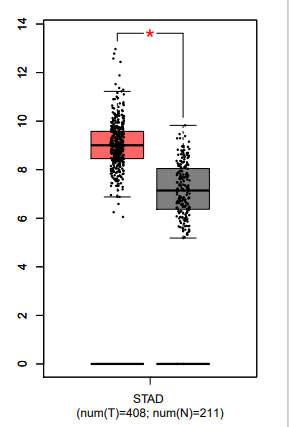
 CTSB
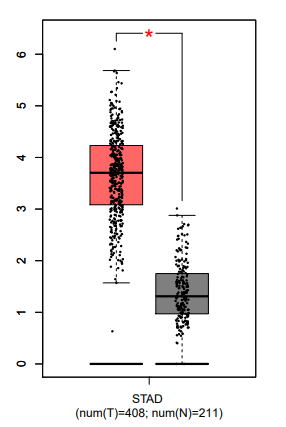
CHEK1
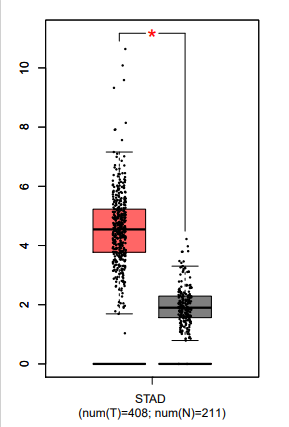
 MET
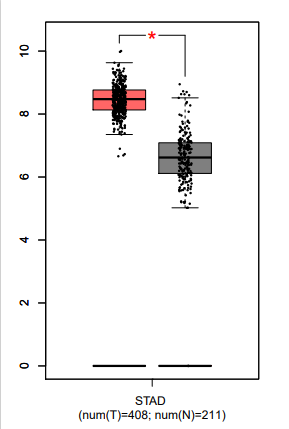
 RAC1
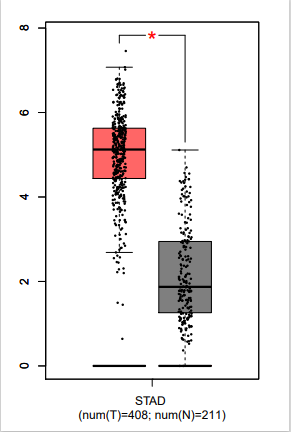
 PLK1
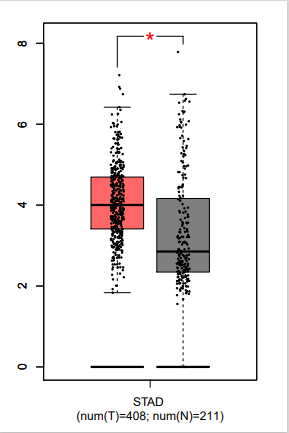
 TGFBR


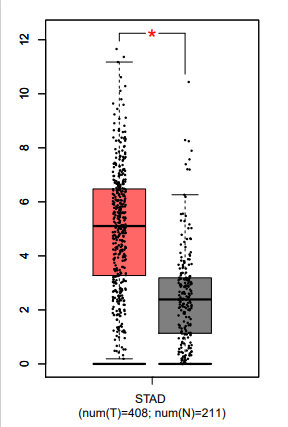
 MMP1
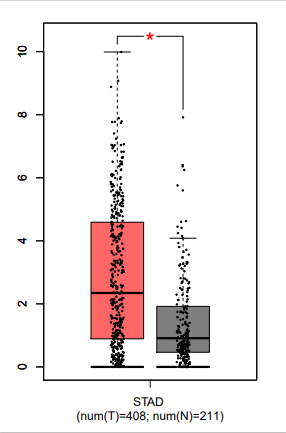
MMP3
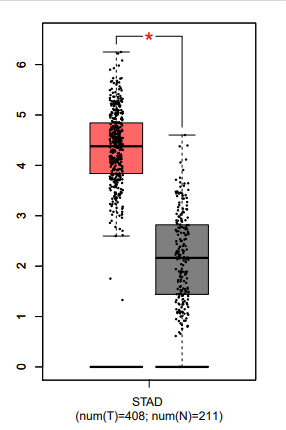
 DHFR
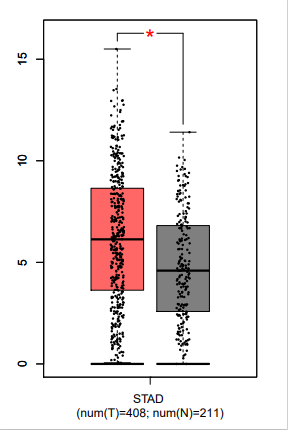
 PLA2G2A

**Supplementary Figure 3.** Kaplan-Meier survival curves analysis of 63 ISO gastric cancer target genes (the picture above shows 25 genes with survival differences).


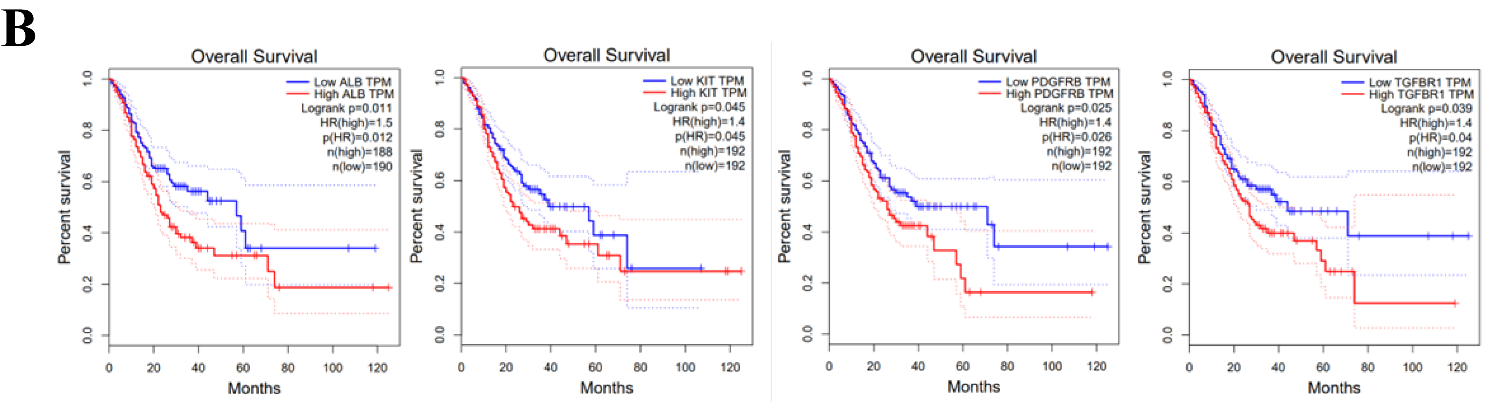


**Supplementary Figure 4.** Normal/cancer comparison of 25 genes with survival differences among the 63 ISO gastric cancer target genes (obtaining four genes with survival differences and differential expression ALB, KIT, PDGFRB and TGFBR1).
